# Transcriptome Deconvolution Reveals Absence of Cancer Cell Expression Signature in Immune Checkpoint Blockade Response

**DOI:** 10.1158/2767-9764.CRC-23-0442

**Published:** 2024-06-26

**Authors:** Yu Amanda Guo, Tanmay Kulshrestha, Mei Mei Chang, Irfahan Kassam, Egor Revkov, Simone Rizzetto, Aaron C. Tan, Daniel S.W. Tan, Iain Beehuat Tan, Anders J. Skanderup

**Affiliations:** 1 Genome Institute of Singapore (GIS), Agency for Science, Technology and Research (A*STAR), 60 Biopolis Street, #02-01 Genome, Singapore 138672, Republic of Singapore.; 2 School of Computing, National University of Singapore, Computing 1, 13 Computing Drive, Singapore 117417, Republic of Singapore.; 3 Department of Medical Oncology, National Cancer Centre Singapore, Singapore 169610, Republic of Singapore.

## Abstract

Immune checkpoint therapy (ICB) has conferred significant and durable clinical benefit to some patients with cancer. However, most patients do not respond to ICB, and reliable biomarkers of ICB response are needed to improve patient stratification. Here, we performed a transcriptome-wide meta-analysis across 1,486 tumors from ICB-treated patients and tumors with expected ICB outcomes based on microsatellite status. Using a robust transcriptome deconvolution approach, we inferred cancer- and stroma-specific gene expression differences and identified cell-type specific features of ICB response across cancer types. Consistent with current knowledge, stromal expression of *CXCL9*, *CXCL13*, and *IFNG* were the top determinants of favorable ICB response. In addition, we identified a group of potential immune-suppressive genes, including *FCER1A*, associated with poor response to ICB. Strikingly, PD-L1 expression in stromal cells, but not cancer cells, is correlated with ICB response across cancer types. Furthermore, the unbiased transcriptome-wide analysis failed to identify cancer-cell intrinsic expression signatures of ICB response conserved across tumor types, suggesting that cancer cells lack tissue-agnostic transcriptomic features of ICB response.

**Significance:**

Our results challenge the prevailing dogma that cancer cells present tissue-agnostic molecular markers that modulate immune activity and ICB response, which has implications on the development of improved ICB diagnostics and treatments.

## Introduction

Immune checkpoint blockade (ICB) therapy is an established class of immunotherapy that induces significant tumor shrinkage and long-term disease control in many cancers. However, only a subset of patients benefit from ICB, with response rates ranging from 0% to 3% in pancreatic cancer (1) to about 40% in melanoma and microsatellite instable (MSI) tumors (2). Currently, the FDA-approved biomarkers for ICB are PD-L1 expression (3), microsatellite instability (4), and high tumor mutation burden (TMB) of >10 mutations/Mb. (5) While PD-L1 expression is an obvious biomarker for PD-1/PD-L1 blockade therapies, it is only weakly predictive of treatment response in many solid cancer types (6). TMB is a measure of tumor immunogenicity, where increased mutation load is associated with higher neoantigen burden and antitumoral immune responses. However, the predictive power of TMB varies significantly by cancer type and it is difficult to define a single TMB threshold across cancer types (7, 8). Likewise, MSI tumors are more immunogenic, as defective mismatch repair results in large numbers of insertions and deletions in the cancer genomes. Although MSI status is approved as a tissue-agnostic biomarker of ICB response, it is limited by most solid tumor types not displaying microsatellite instability (9, 10).

Transcriptional signatures of tumor immune phenotypes (11–13) and tumor microenvironment (TME) subtypes (14, 15) have been proposed to predict for ICB response. However, many previous analyses were limited to annotated cancer- or immune-related genes (11, 12, 14, 15) or to single cancer types (13). Furthermore, analysis of large-scale ICB trial data found that existing transcriptional signatures often have limited predictive accuracy in new patient cohorts (6). Two recent meta-analyses investigated the genomic and transcriptomic predictors of ICB response (16, 17). Litchfield and colleagues (16) evaluated previously described biomarkers of ICB response, and found TMB and *CXCL9* expression as the most consistent predictors of ICB response.

Bareche and colleagues performed a comparative analysis of existing gene signatures of ICB response, and proposed a new predictive gene signature (17). However, existing large-scale studies have been restricted to bulk tumor transcriptomic data, providing limited insights into the roles of cancer and stroma cells in the TME during ICB therapy.

Activation of effector immune cells such as T cells and natural killer (NK) cells are dependent on a balance of costimulatory and coinhibitory interactions between these effector cells, antigen-presenting immune cells such as dendritic cells and macrophages, as well as cancer cells in the TME. Dysregulation of these ligand–receptor interactions, or immune checkpoints, leads to immunosuppression in the tumor. However, it is challenging to tease apart these complex interactions between cancer and immune cells using bulk tumor transcriptomics. For example, although inhibitory immune checkpoint ligand PD-L1 is a known biomarker of ICB response, it is unclear whether cancer cells escape immune cell killing by expressing PD-L1, or recruiting PD-L1^+^ immune cells, with studies supporting both scenarios (18). Therefore, learning the cell-type specificities of ICB biomarkers can provide insights on the underlying mechanisms of immune evasion and ICB resistance. While single-cell transcriptomics has been applied to explore immune cell types and molecular mechanisms associated with ICB response, single-cell transcriptomics studies are currently limited to small patient cohorts (19, 20). The small sample size, coupled with high intrapatient and interpatient heterogeneity and intrinsic noise in single-cell data, limits the power of single-cell transcriptomics for systematic biomarker discovery.

To study features of ICB response in cancer cells as well as in their neighboring stromal (nonmalignant) cells, we applied a transcriptome deconvolution technique to estimate stroma-specific and cancer cell–specific expression of individual genes from bulk tumor transcriptomes. We used a robust and validated non-negative least squares (NNLS) approach (21), which have previously been applied across 8,000 The Cancer Genome Atlas (TCGA) tumors from 20 solid cancer types to uncover ligand-receptor cross-talk and metabolic states of cancer and stroma cells in the TME (22, 23). Here, we applied this transcriptome deconvolution technique to study the differential expression signatures of ICB responders and nonresponders in cancer and stroma cells. Using a cohort of 1,486 tumors with clinically annotated ICB response and tumors with expected ICB outcomes based on microsatellite status, our analysis revealed a lack of cancer cell–intrinsic gene expression signatures associated with ICB response across tumor types. In contrast, in stromal cells, we identified multiple immunomodulators and checkpoint genes recurrently associated with ICB response across cohorts and tumor types. Many of these genes have previously been linked with ICB response, and we uncovered a subset of novel and potentially immune-suppressive genes negatively correlated with response. Using these conserved stromal-specific gene signatures, we developed a multivariate classifier of ICB response that demonstrated improved accuracy over existing biomarkers in unseen patient cohorts and tumor types.

## Materials and Methods

### Clinical Cohorts Treated with ICB

Data from the following studies were used for discovery:

- Kim and colleagues (24) cohort comprises 45 patients with metastatic gastric cancer treated with PD-1 inhibitor pembrolizumab
- Mariathasan and colleagues (25) cohort comprises 298 patients with metastatic urothelial cancer treated with PD-L1 inhibitor atezolizumab
- Liu and colleagues (26) comprises 118 patients with metastatic melanoma treated with PD-1 inhibitors nivolumab or pembrolizumab
- Gide and colleagues (27) comprises 73 patients with metastatic melanoma treated with anti-PD-1 alone (nivolumab or pembrolizumab), or with anti-CTLA-4 (nivolumab or pembrolizumab with ipilimumab)

Data from the following studies were used for validation:

- Riaz and colleagues (28) comprises 49 patients with advanced melanoma treated with PD-L1 inhibitor nivolumab.
- Pender and colleagues (29) comprises 87 metastatic tumors from 20 solid cancer types treated with a mix of ICB agents.
- Freeman and colleagues (30) comprises 31 patients with advanced melanoma treated a mix of ICB agents.
- Jung and colleagues (31) comprises 27 patients with non–small cell lung cancer treated PD-1 or PD-L1 inhibitor.
- Nathanson and colleagues (32) comprises 19 patients with melanoma treated CTLA-4 inhibitor ipilimumab.
- Ratovomanana and colleagues (33) comprises 24 patients with metastatic MSI colorectal cancer treated nivolumab alone or in combination with ipilimumab.

RNA sequencing (RNA-seq) data are available for all study cohorts, while exome sequencing data are only available for the Kim and colleagues study (24).

### Gene Expression Data Collection and Normalization

Expression data of TCGA colon adenocarcinoma (COAD), rectal adenocarcinoma (READ), stomach adenocarcinoma (STAD), and endometrial tumors were downloaded from the UCSC Xena repository [https://xenabrowser.net/datapages/?cohort=TCGA%20Pan-Cancer%20(PANCAN)&removeHub=https%3A%2F%2Fxena.treehouse.gi.ucsc.edu%3A44]. Raw transcriptomic data of Kim and colleagues (24) gastric ICB cohort were downloaded from the European Nucleotide Archive, and processed using the bcbio-nextgen pipeline. Briefly, RNA-seq reads were aligned to the human transcriptome using STAR (34), and transcripts per kilobase million (TPM) counts were quantified using SALMON (35). For all other ICB studies, summarized transcriptomic data were obtained from the original publications. Fragments per kilobase million (FPKM) data from Nathanson and colleagues (32) and count data Ratovomanana and colleagues (33) were converted to TPM. For all other ICB cohorts, TPM data were directly downloaded from the original publications. Genes with 0 expression in more than 50% of the samples were removed. All gene expression data were log_2_ transformed and upper quantile normalized across all cohorts.

### Tumor Purity Estimation

Purity estimates of TCGA tumors were obtained from a previous publication (22), where consensus purity was estimated from mutation allele frequencies, copy-number variations, and RNA profiles of each tumor. As raw genomic sequencing data were unavailable for all ICB cohorts except for Kim and colleagues (36), tumor purity of samples without genomic data were estimated using PUREE (37), a machine learning method for tumor purity estimation from bulk RNA expression data. For the Kim and colleagues gastric ICB cohort (36), where genomic data were also available, we calculated consensus purity estimates based on both genomic and transcriptomic data.

We first computed purity estimates using two RNA-based methods [PUREE (37) and Estimate (38)], and two DNA-based methods [Sequenza (39), PurBayes (40)]. Then, we performed quantile-quantile normalization on the purity estimates of individual methods, and took the mean normalized purity estimate as the consensus purity.

### MSI Status and ICB Response Classification

MSI status of TCGA tumors were obtained from two previous pan-cancer studies of microsatellite instability (9, 10). Epstein–Barr virus (EBV) status of TCGA STAD tumors was obtained from a previous TCGA study on gastrointestinal cancers (41). Clinical annotations of ICB response based on the RECIST were obtained from the original studies. Consistent with recent literature (16, 25), we classified complete responders and partial responders as responders and stable disease and progressive disease as nonresponders.

### Expression Deconvolution

We modeled bulk tumor expression as the sum of cancer and stroma expression, weighted by cancer purity (22). This can be written as:



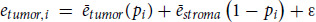



where *e_tumor,i_* is the bulk tumor expression for the gene in sample *i*, *ē_tumor_* and *ē_stroma_* are the average cancer- and stroma-specific expression, *p_i_* denotes cancer purity for sample *i*, and ɛ is the residual. Given bulk expression (*e_tumor_*) and tumor purity (*p*), NNLS regression can be used to estimate cancer- (*ē_tumor_*) and stroma-specific (*ē_tumor_*) expression for each gene. The SE of the predicted mean expression of compartment *h* is calculated as:



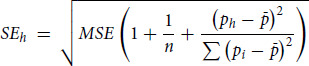



Where *n* is the sample size, 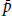 is the mean tumor purity over all samples, *p_h_* = 1 for cancer-specific expression, and *p_h_* = 0 for stroma-specific expression; *MSE* is the mean squared error.

### Permutation Statistic to Identify Differentially Expressed Genes

To test differences in cancer (or stroma) expression between two response groups (e.g., *ē_tumor, R_ – ē_tumor, NR_*), the above approach is applied to each group separately, and a test statistic is calculated as:



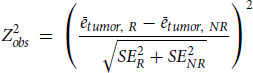



To assess significance under the null hypothesis of no difference in cancer (stroma) expression between the two response groups, a null distribution is generated by permutation, where group labels are first randomly shuffled 10,000 times and the above procedure is applied to each permuted dataset, generating a null distribution of 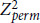 statistics. A *P*-value is calculated as:



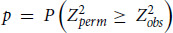



That is, the probability of observing 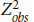 or larger, given the null hypothesis is true.

### Identification of Consensus Differentially Expressed Genes Across Cancer Types

For each gene and each tumor compartment (caner or stroma), a meta *P*-value is computed by combining the permutation *P*-values of all cancer cohorts using Fisher method. We used the Bonferroni correction to adjust for multiple testing to maintain the overall α at 0.01. Genes that are differentially expressed in different directions in at least two cohorts were removed. Finally, to eliminate differentially expressed genes (DEG) that are involved in MSI biology but are not directly associated with ICB response, we require the final list of consensus DEGs to be differentially expressed in at least one ICB cohort (nominal *P*-value <0.1).

### Pathway Enrichment Analysis

To find out whether the DEGs identified are enriched in specific biological processes, pathway enrichment analysis was performed using ClueGo (42) with default parameters. Briefly, two-sided-hypergeometric statistic was used to compute the enrichment of a given list of DEGs in Gene Ontology (GO) biological processes terms, and the resulting *P*-values were corrected for multiple testing using the Bonferroni step down approach. To aid interpretation, significant biological terms with high overlap as measured by the kappa coefficient were merged into functional groups. To calculate the enrichment of stroma DEGs in immune-related functions, we defined immune-related genes as genes in the “immune system process” term and its child terms in the GO biological processes dataset. In addition, we manually curated stroma DEGs not labeled as immune genes by GO, and further labeled *FCER1A*, *GZMH*, *FCRL6*, *SIRPG*, *JAKMIP1*, and *WARS* as immune-related genes based on literature search.

### Analysis of Immune Cell Expression Data from the Human Protein Atlas

From the Human Protein Atlas, we downloaded the normalized gene expression of 81 cell types from 31 single-cell RNA-seq (scRNA-seq) datasets, and the normalized gene expression of 18 immune cell types isolated with cell sorting. Then, we plotted the log_2_ transformed expression of the candidate stromal DEGs in individual immune cell types.

Download links for Human Protein Atlas data:

https://www.proteinatlas.org/download/rna_single_cell_type.tsv.zip
https://www.proteinatlas.org/download/rna_immune_cell.tsv.zip

### Analysis of scRNA-seq Data

Gene-level normalized counts from Bi and colleagues (43) were downloaded from the Single Cell Portal. A total of 34,327 cells from eight renal cell carcinoma tumors were analyzed, of which 17,672 cells from five tumors were from ICB-treated patients. Cells with low library size and high mitochondrial contamination were removed. For each annotated cell type, we calculated the average expression and proportion of expressing cells for each DEG, separately for cells from ICB responders and nonresponders. To generate the doplot in Fig. 3A, we scaled the expression counts of each gene using z-score normalization, and computed the average scaled expression and the prevalence of expression of each gene across all stromal (noncancer) cell types.

### Ligand-Receptor Analysis

We curated 30 immune checkpoint ligand-receptor pairs, their cell type specificity and the likely effect of the checkpoint interaction (activating or inhibitory) from literature review. In addition, we curated 25 cytokine/chemokine ligand-receptor pairs involved in immune regulation. To identify ligand-receptor pairs with coordinated expression changes between ICB responders and nonresponders, we surveyed all ligand-receptor genes with nominal *P*-value >0.1 from the differential gene expression permutation test.

### Predictive Modeling of ICB Response

The *IOSelect* immune score is defined as the ratio of the mean expression of the top 10 DEGs upregulated in ICB responders (*CXCL9*, *CXCL13*, *IFNG*, *KLRD1*, *GBP1P1*, *CRTAM*, *FASLG*, *GBP5*, *CD8A*, *PRF1*) to the mean expression of all eight DEGs downregulated in ICB responders (*FCER1A*, *PID1*, *CH25H*, *NPR3*, *PREX2*, *NPR1*, *FAM134B*, *SDPR*). To build the *IOselect* model, we used 461 patients from three discovery cohorts [Mariathasan and colleagues (25), Liu and colleagues (26), Kim and colleagues (24)] with both RNA-seq and TMB as training data.

Whole-exome sequencing FASTQ files of Kim and colleagues (24) gastric ICB cohort were downloaded from the European Nucleotide Archive, and processed using the bcbio-nextgen pipeline. Somatic mutations were called by VarScan (44), MuTect (45), VarDict (46), and FreeBayes (47) using default parameters of the bcbio-nextgen pipeline. The final set of mutations was called using an ensemble mutation caller, SMuRF (48), from the outputs of the four mutation callers in the bcbio-nextgen pipeline. TMB was calculated as the number of nonsynonymous mutations per megabase. For the Mariathasan and colleagues (25) and Liu and colleagues (26) cohorts, TMB was obtained from the original publications. All TMB values were standardized using z-score transformation (mean = 0, SD = 1). We fitted a logistic regression model using *IOSelect* immune score and TMB as input features.

Next, we examined whether we can build a more compact model with comparable performance. Using the same training data, we applied the least absolute shrinkage and selection operator (LASSO) to select top features from the 59 stroma DEGs and TMB. LASSO penalizes the sum of the absolute values of the regression coefficients, shrinking some coefficients to zero, and thereby identifies a simpler and more interpretable model. The regularization parameter λ was chosen by 10-fold cross-validation such that the error of the selected model was within 1 SD from the minimum error. LASSO regression and cross-validation were performed using the “glmnet” package in R. To select for the most robust features, we bootstrapped 100 samples with 90% of the data in each bootstrap, and selected features that are found by at least 75% of the bootstraps for the final model. We found that TMB, *IFNG*, and *FCER1A* are the most stably selected features, and built the logistic regression model on all 461 training samples using these three features.

### Construction of ICB Response Prediction Model Using Cancer DEGs

To evaluate whether the cancer-cell specific DEGs identified are predictive of ICB response in independent cohorts, we fitted a logistic regression model using all nine cancer-intrinsic DEGs (*RPL22L1*, *PGAM1*, *RPS27L*, *TIMM21*, *COPS3*, *COPS4*, *ZNF717*, *TMEM176A*, and *SMAP1*). We used patients from all four discovery ICB cohorts as training data and evaluated the performance of the cancer DEG model on six independent test cohorts described in the previous section.

### Benchmarking ICB Prediction Model Against Existing Biomarkers

T-cell inflamed gene-expression profile (GEP) scores were calculated from normalized RNA-seq data using the weights provided by Ayers and colleagues 2018 (11). TIDE scores were calculated from normalized RNA-seq data using the TIDE web platform (49). The *CXCL-9*+TMB model was trained on our training cohort using logistic regression. The cytolytic score is calculated as the geometric mean of *GZMA* and *PRF1* expression (50). Vigex score is computed as the mean of z-score scaled expression of 12 genes (*CXCL9*, *CXCL10*, *CXCL11*, *IFNG*, *PRF1*, *IL7R*, *GZMA*, *GZMB*, *PDCD1*, *CTLA4*, *CD274*, *FOXP3*; ref. 51).

### Immune Cell Enrichment Analysis

Immune deconvolution was performed using CIBERSORTx (with the LM22 signature matrix; ref. 52) to estimate the absolute abundance of immune cell subsets for each tumor. Immune cell subsets that are differentially infiltrated between responders and nonresponders were identified using the Wilcoxon rank-sum test.

### Data and Code Availability

The code used to generate figures in the article is provided in Supplementary Data S1. The transcriptomic and clinical data of the ICB cohorts were obtained from the original publications (Supplementary Table S3).

## Results

### Patient Cohorts Used for Transcriptome-wide Analysis of ICB Response

**FIGURE 1:**
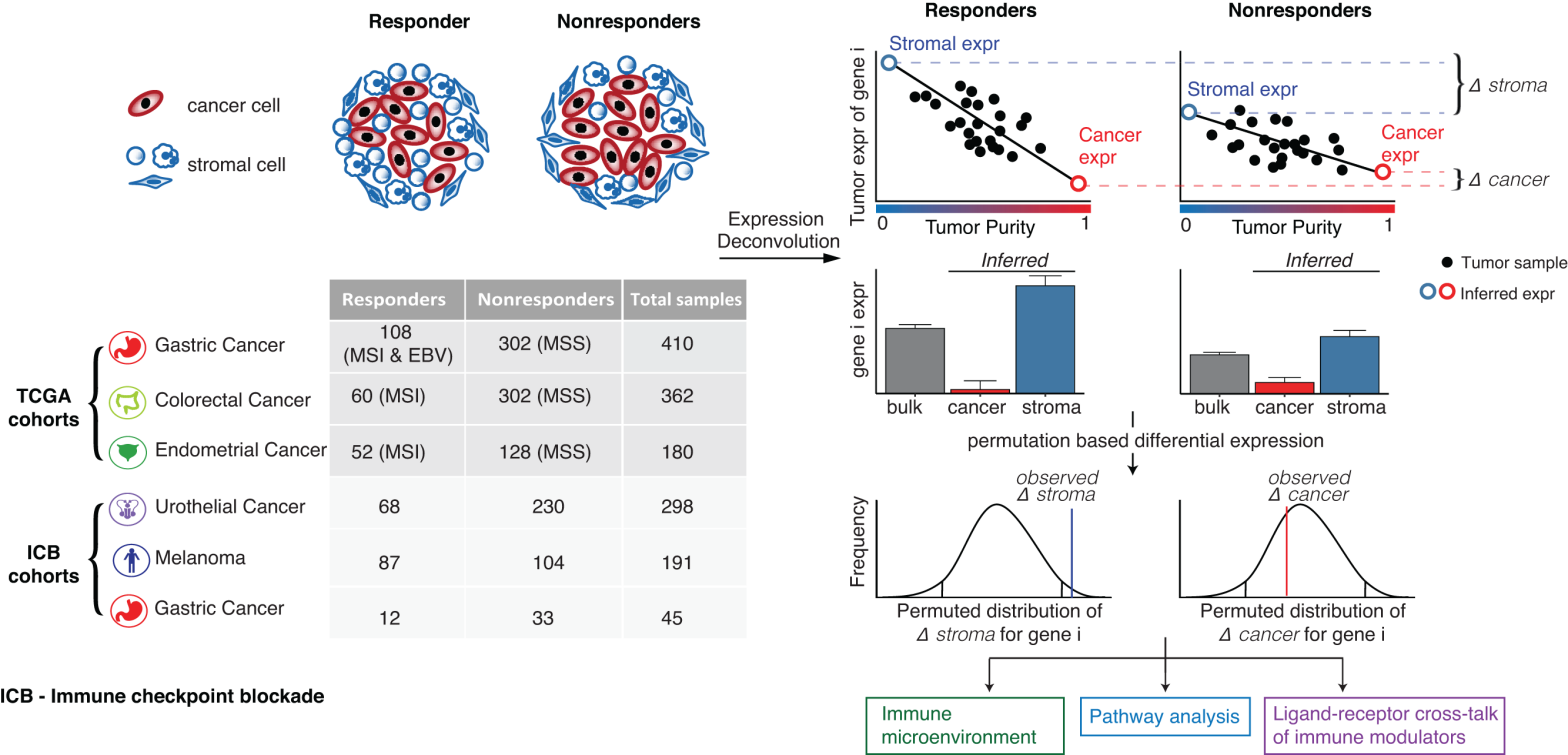
Data summary and schematic of workflow for the analysis of determinants of immunotherapy response.

To systematically explore biomarkers of ICB response transcriptome-wide, we obtained RNA-seq data for 534 pretreatment tumors from ICB-treated patients, comprising four studies of three tumor types [gastric cancer (24), urothelial cancer (25), and melanoma (26, 27); Fig. 1; Supplementary Table S1, and Materials and Methods]. Although microsatellite instability is not a direct measure of ICB efficacy, MSI tumors are highly enriched for ICB responders. Depending on the cancer type, about 30%–50% of MSI tumors respond to ICB, compared with <10% for microsatellite stable (MSS) tumors (2, 53, 54). Adopting a semisupervised learning approach, we considered MSI tumors as ICB responder-enriched, and MSS tumors as nonresponder-enriched, in our meta-analysis for discovery of putative biomarkers of ICB response. We obtained the transcriptomic profiles of 952 tumors from three tumor types in TCGA with the highest frequency of MSI tumors (colorectal cancer, gastric cancer, and endometrial cancer, Fig. 1). As EBV-positive gastric tumors are also enriched for ICB responders (24, 54), we grouped MSI and EBV tumors together as the responder-enriched group for gastric cancer. In total, the discovery cohort comprised of 1,486 tumors from five cancer types.

### Tumor Transcriptome Deconvolution to Identify Features of ICB Response in Cancer and Stromal Cells

We performed tumor transcriptome deconvolution to estimate the stromal- and cancer-cell expression of each gene in tumors from responders (MSI) and nonresponders (MSS; Fig. 1). Briefly, we first used a consensus approach to infer tumor purities based on transcriptomic and genomic data (where available, Materials and Methods). For each ICB response group, we estimated gene expression levels in stromal and cancer cells using a NNLS regression approach (22, 23). Finally, we identified cancer/stromal cell DEGs between ICB responders and nonresponders using a permutation-based statistic (Fig. 1; Supplementary Fig. S1). To evaluate the robustness of the deconvolution approach in the ICB cohorts, we confirmed that known lineage-specific genes showed the expected expression differences between cancer and stroma compartments (Supplementary Fig. S2).

### Top Stromal Genes Associated with ICB Response are Enriched in Immune Pathways

**FIGURE 2:**
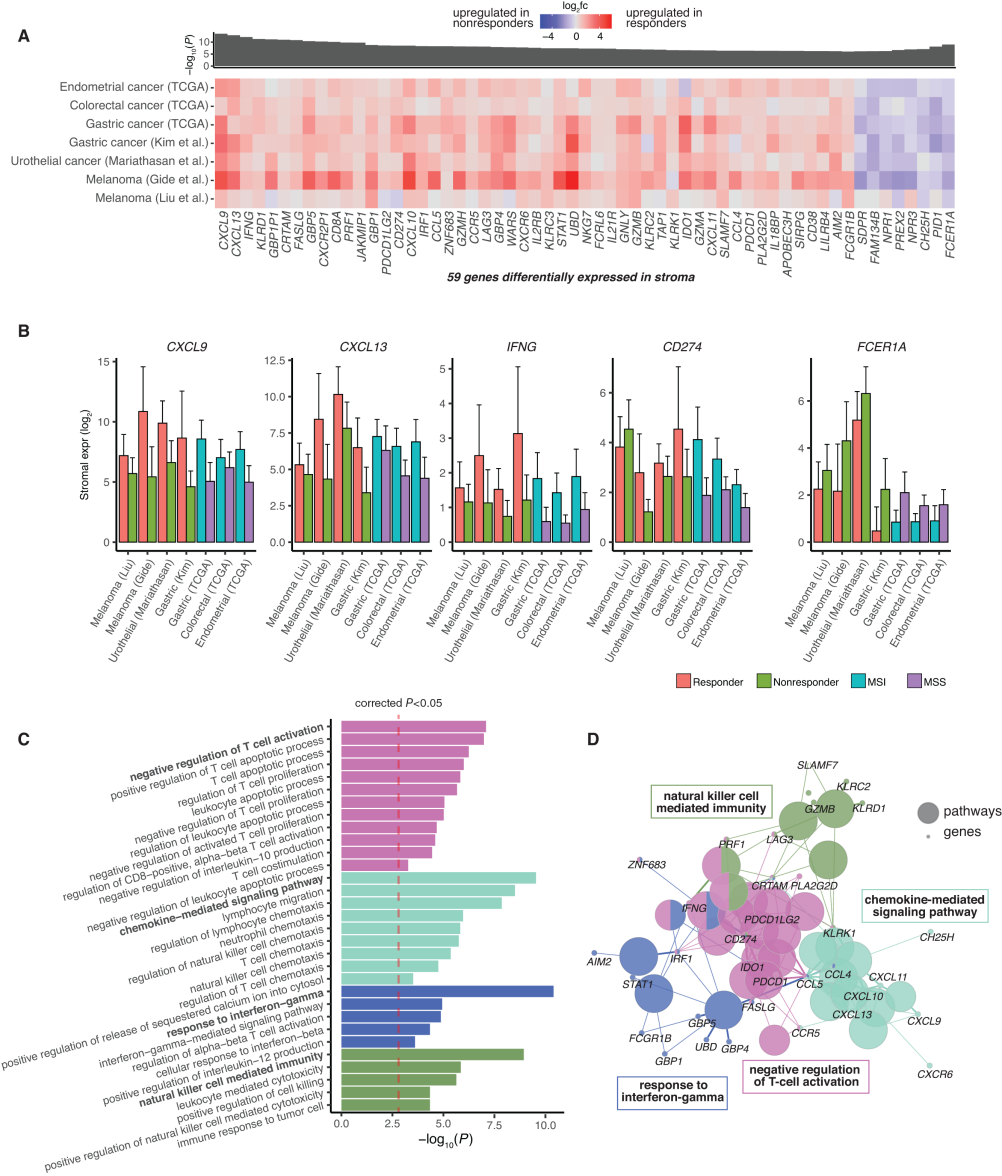
DEGs and pathways between responders and nonresponders in the stroma. **A,** Heat map of genes consistently differentially expressed across discovery cohorts in the stroma. Heat maps are colored by the log_2_ fold change of gene expression. *P*-values of individual cohorts are combined using the Fisher method, and the −log_10_(meta *P*-value) is presented in the bar plot. Genes with *q*-value <0.01 and *P*-value <0.1 at least one ICB cohort are shown. **B,** Bar plots of deconvoluted stromal expression of the top DEGs. Error bars represent estimated SE. **C,** Pathway enrichment of DEGs in the stroma. Bar plot shows the −log_10_(*P*-value) of significantly enriched pathways. Similar pathways are grouped by color. **D,** Network representation of enriched pathways. Large nodes represent enriched pathways. Pathways with high similarity are fused and shown in the same color. Small nodes represent genes associated with each pathway.

From this unbiased transcriptome-wide analysis, we identified 59 genes with differential expression in stromal cells across all cohorts and tumor types (Fig. 2A and B; Supplementary Fig. S3A and S3C, Materials and Methods). To ensure we identify genes that are directly associated with ICB response instead of MSI biology-related genes, we only considered genes that are differentially expressed in at least one ICB cohort. The stroma DEGs identified tended to show consistent differential expression across both MSI/MSS cohorts as well as the four ICB cohorts (Fig. 2A). Among these DEGs, 51 (86%) had immune-related functions as defined by the GO (see Materials and Methods), significantly higher than random expectation (*P* < 2.2e-16, Fisher exact test). Stromal DEGs include genes involved in immune checkpoint signaling (e.g., *CD274*, *LAG3*, *CTLA4*), IFNγ signaling (e.g., *IFNG*, *IRF1*, *STAT1*), T-cell effector function (e.g., *PRF1*, *GZMA*, GZMB), and NK cell–mediated cytotoxicity (*KLRD1*, *KLRC2*). Next, we performed pathway analysis to quantify the enrichment of specific biological functions among the stromal DEGs associated with ICB response. We identified 31 enriched GO biological process terms, all of which were immune related (Fig. 2C). These 31 terms could be further grouped into four clusters based on similarity (see Materials and Methods): T-cell activation, chemokine signaling, IFNγ response, and NK cell–mediated cytotoxicity (Fig. 2D).

The top two DEGs with the greatest overexpression in ICB responders were chemokines *CXCL9* and *CXCL13*. *CXCL9* is responsible for T-cell trafficking through binding the *CXCR3* receptor on T cells and has been associated with increased T-cell infiltration in tumors (55). *CXCL13* is involved in both B-cell and T-cell migration via binding to the *CXCR5* receptor. Production of *CXCL13* by T follicular helper (Tfh) cells is essential for tertiary lymphoid structure (TLS) formation at tumor sites as well as germinal center B-cell activation (56), which has been linked with antitumoral response to ICB (57). Consistent with our meta-analysis, recent studies have also proposed *CXCL9* and *CXCL13* as key predictors of improved ICB response (58, 59). Remarkably, these two chemokines were also highlighted by a recent meta-analysis that evaluated existing biomarkers of ICB response (16), suggesting that *CXCL9* and *CXCL13* are indeed the strongest individual transcriptomic predictors of ICB response independent of tumor type.

### Stromal Genes Negatively Associated with ICB Response

Although high immune infiltration is generally associated with better ICB response (11, 50, 60), our analysis uncovered a group of immune-related genes consistently associated with poor ICB outcomes across tumor types. Among these genes, *FCER1A* showed the strongest and most consistent overexpression in ICB nonresponders. *FCER1A* encodes a subunit of the IgE receptor. While *FCER1A* is an established marker of basophils, mast cells, and dendritic cells, it is also expressed on immunosuppressive M2 macrophages (61) and tumor-associated macrophages (TAM; ref. 62). *CH25H*, a gene encoding enzyme cholesterol 25-hydroxylase that catalyzes the formation of 25-hydroxycholesterol (25-HC), was also upregulated in tumors of ICB nonresponders. Interestingly, 25-HC is produced by macrophages in response to type-I IFN signaling and has numerous immunologic effects including B-cell chemotaxis, macrophage differentiation, and regulation of inflammatory response (63). We also observed two natriuretic peptide receptors, NPR1 and NPR3, among genes upregulated in ICB nonresponders. Natriuretic peptides are vasoactive peptides that have been shown to regulate vascular remodeling and promote angiogenesis (64).

### scRNA-seq Confirms Differential Expression of the Stromal Biomarkers in Specific Immune Cell Subsets

**FIGURE 3:**
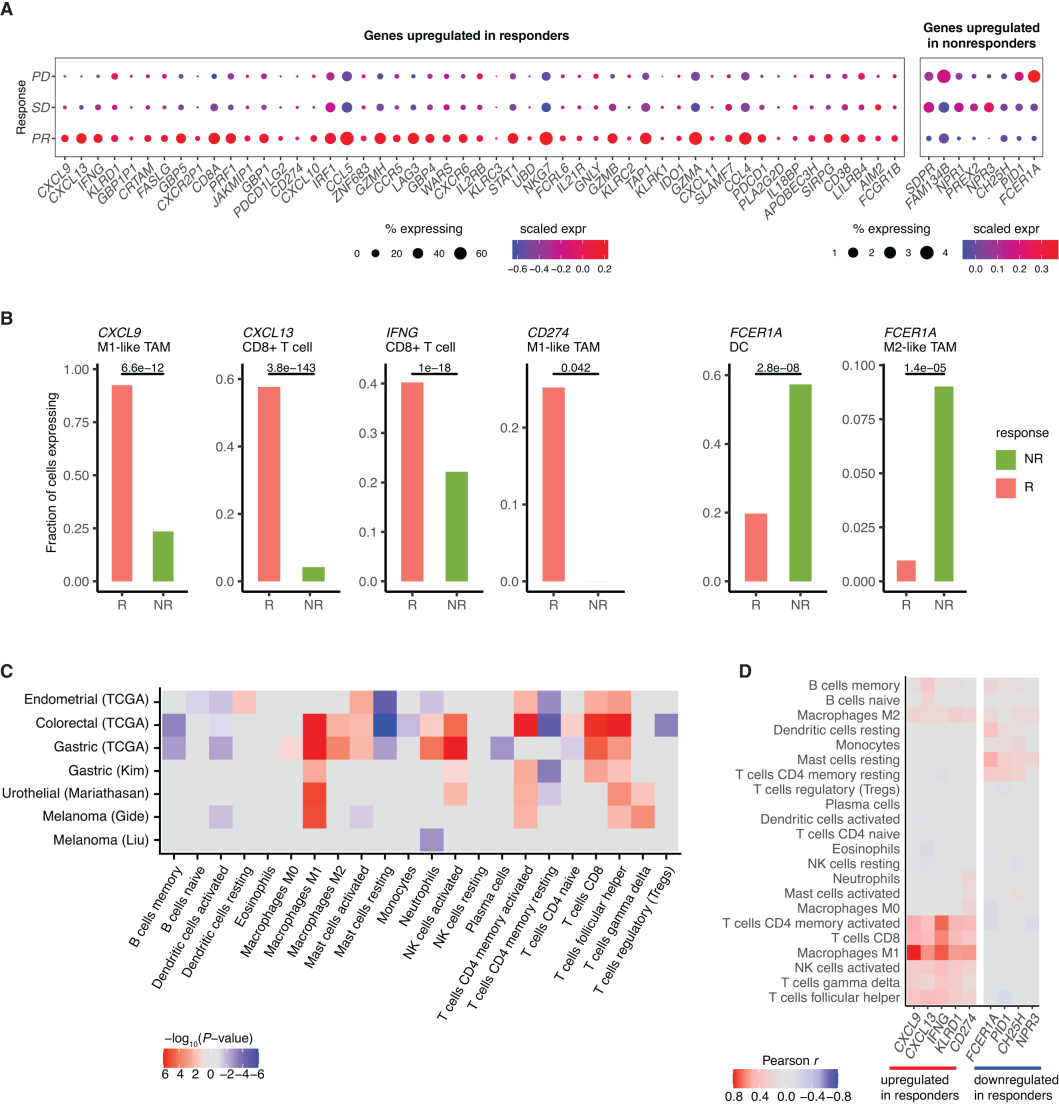
Validation and characterization of stromal DEGs with scRNA-seq and immune cell deconvolution analysis. **A,** Dotplot showing the percentage of stromal (nonmalignant) cells expressing each gene for each response category. Color represents the average scaled expression of the gene in stromal cells (PD, progressive disease; SD, stable disease; PR, partial response). **B,** Cell-type specific expression of the top stromal biomarkers in ICB responders (PR) and nonresponders (PD/SD). **C,** Heat map of the association between immune cell types and ICB response. Abundance of immune cell types of each tumor is estimated using CIBERSORTx. Heat map is colored by the signed −log_10_(*P*-value) of the difference in CIBERSORTx scores between responders (MSI) and nonresponders (MSS). Cell types with no significant difference in abundance between ICB responders and nonresponders (*P*-value >0.1) were colored gray. **D,** Consensus correlations between the abundance of immune cell types and expression of the top stromal biomarkers. Mean Pearson correlation coefficients across the seven discovery cohorts were plotted for each cell type-gene pair. Positive correlations were colored red, negative correlations were colored blue, and tiles with Pearson correlation coefficient <0.1 were colored gray.

Using a scRNA-seq dataset of patients with renal cell cancer treated with ICB (43), we investigated the expression of the stromal DEGs in specific stromal cell subsets and their association with ICB response. First, we confirmed that the stromal DEGs were differentially expressed between ICB responders and nonresponders in stromal (noncancerous) cells (Fig. 3A). Next, we examined the expression distribution of the stromal DEGs among individual cell types. We found that *CXCL9* and *CD274* were most highly expressed in M1-like TAMs, while *CXCL13* and *IFNG* were enriched in CD8^+^ T cells (Supplementary Fig. S4). Among DEGs associated with poor ICB response, *FCER1A* was most highly expressed in dendritic cells, mast cells, and M2-like TAMs. *PID1* and *CH25H* were enriched in myeloid cells and fibroblasts, while *NPR3*, *NPR1*, *PREX2*, and *SDPR* were specifically expressed by endothelial cells (Supplementary Fig. S4). Thus, the stroma DEGs likely encompasses multiple signatures of the TME, including T-cell infiltration, macrophage polarization, angiogenesis as well as fibroblast abundance. Using orthogonal expression data of immune cells subtypes from flow cytometry and scRNA-seq experiments from the Human Protein Atlas, we confirmed the cell-type specific expression of the top stromal DEGs (Supplementary Fig. S5, Materials and Methods). Furthermore, we found that the differential gene expression signal between ICB responders and nonresponders was associated with increased abundance of specific immune cell subsets. For example, the CXCL9+ M1-like TAMs and CXCL13+ T cells were more prevalent in ICB responders than in nonresponders. In contrast, FCER1A+ dendritic cells and FCER1A+ M2-like TAMs were more prevalent in nonresponders (Fig. 3B). Overall, these results suggest that the stromal DEGs represent specific components of the TME that associate with tumor response to ICB.

### Immune Cell Subtype Analysis Identifies M1 Macrophages as Strong Correlate of ICB Response

To further characterize immune cell populations associated with ICB response, we estimated the immune cell composition of each tumor using CIBERSORTx (52) and compared the abundance of immune cell subsets between ICB responders and nonresponders. M1 macrophages, Tfh, and activated CD4 memory T cells had higher abundance in ICB responders as compared with nonresponders (Fig. 3C; Supplementary Fig. S6). M1 macrophages are known to be proinflammatory and antitumoral (65). A recent study found M1 macrophage infiltration to be predictive to ICB response in urothelial cancer (66). Our results further demonstrate that M1 macrophage infiltration could be a general feature of ICB response across multiple cancer types. Tfh cells are specialized CD4^+^ T cells that aid the formation of germinal centers in TLSs (67), interacting with B cells to activate antibody responses (68) and enhancing CD8^+^ T-cell effector functions (69). While the presence of Tfh cells has been associated with favorable survival in multiple cancer types (57, 68–70), their putative role in ICB response has not been studied. Our analysis also demonstrated that while resting CD4 memory T cells were enriched in nonresponders, activated CD4 memory T cells were enriched in ICB responders. This suggests that preexisting CD4^+^ T-cell immunity is important for effective antitumoral response upon ICB treatment.

Next, we examined associations between the top DEGs and infiltration of specific immune cell subsets. We found that the top gene expression markers of good ICB response were generally associated with abundance of M1 macrophages, CD4^+^/CD8^+^ T lymphocytes, and activated NK cells across the discovery cohorts (Fig. 3D; Supplementary Fig. S7; Supplementary Table S2). In contrast, the DEGs associated with poor ICB response, including *FCER1A*, were correlated with resting state of mast cells, CD4^+^ memory T cells, and dendritic cells, suggesting that these genes are likely markers of a quiescent or immune-suppressive microenvironment (Fig. 3D; Supplementary Fig. S7; Supplementary Table S2).

### Cancer Intrinsic Gene Expression Features of ICB Response Tend to be Tumor Type Specific

**FIGURE 4:**
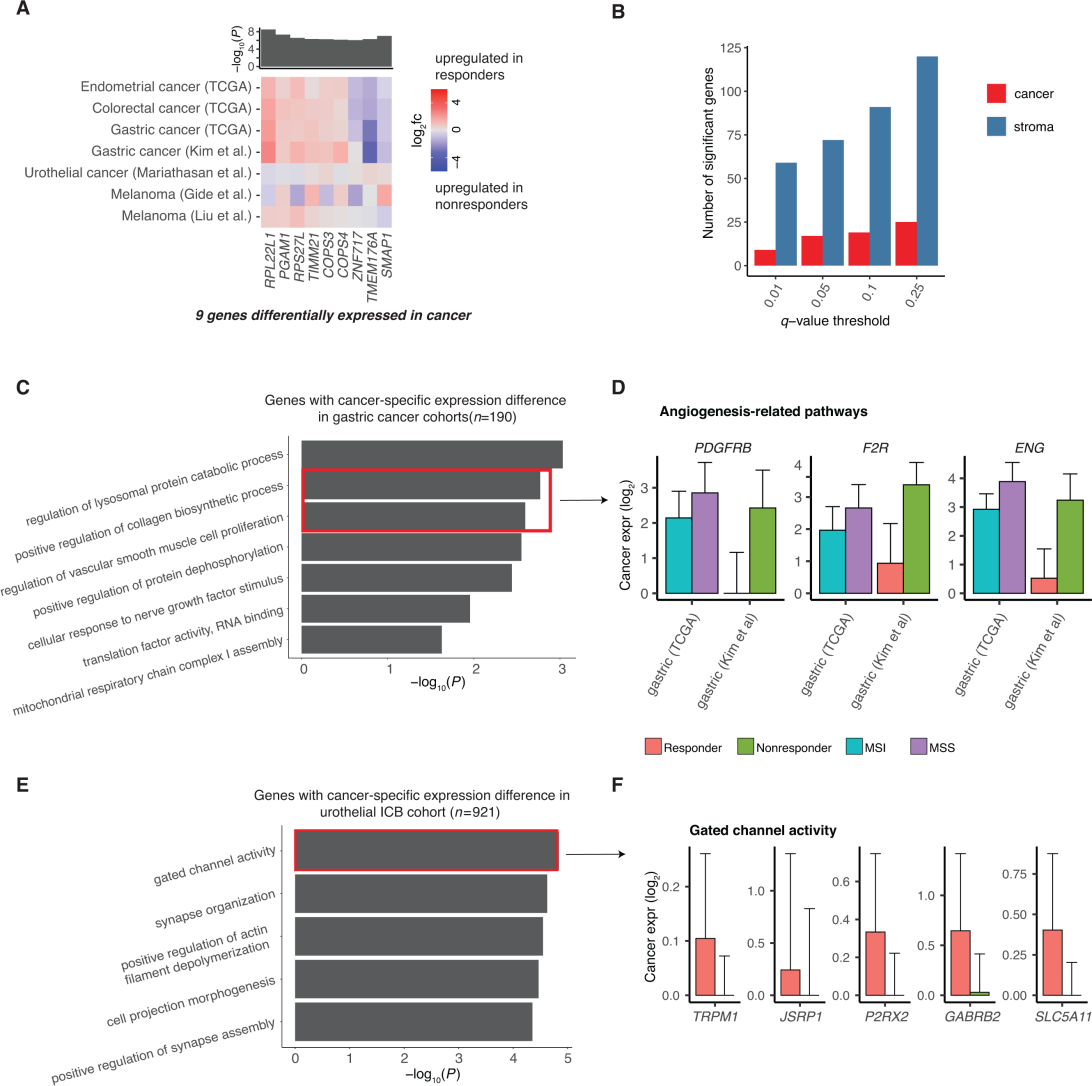
DEGs between responders and nonresponders in the cancer compartment. **A,** Heat map of genes consistently differentially expressed across discovery cohorts in the cancer cells. Heat maps are colored by the log_2_ fold change of gene expression. *P*-values of individual cohorts are combined using the Fisher method, and the −log_10_(meta *P*-value) is presented in the bar plot. Genes with *q*-value <0.01 and *P*-value <0.1 at least one ICB cohort are shown. **B,** Bar plot shows the number of significant genes found in the stroma and cancer compartment at different *q*-value cutoffs. Pathway analysis of genes with cancer-specific expression differences in gastric cancer (**C**) and urothelial cancer (**E**). Bar plots of deconvoluted cancer expression of DEGs in the top enriched pathway in gastric cancer (**D**) and urothelial cancer (**F**). Error bars represent estimated SE.

Previous studies have proposed multiple mechanisms by which cancer cells may directly suppress tumor immunity and impede ICB therapy (18, 71). Surprisingly, our meta-analysis across tumor types revealed a paucity of DEGs associated with ICB response in cancer cells (Fig. 4A and B). In cancer cells, we identified nine genes differentially expressed between ICB responders and ICB nonresponders across tumor types (Fig. 4A; Supplementary Fig. S3B and S3D). However, we observed that the differential expression signal for these nine genes was mostly driven by the MSI ICB responder–enriched cohorts. As compared with stromal DEGs (Fig. 2A), the signal for the cancer DEGs was less consistent across the distinct ICB cohorts (Fig. 4A). Therefore, we hypothesize that the cancer DEGs identified by the meta-analysis are likely related to biology of MSI tumors, rather than general mechanisms of ICB response. Indeed, the nine cancer DEGs were not predictive of ICB response when tested on tumor transcriptomic data from six independent ICB studies (Supplementary Fig. S8). We also confirmed that the paucity of cancer cell DEGs, as compared with stroma cell DEGs, persisted regardless of the significance cutoff in our meta-analysis (Fig. 4B), and we were not able to identify functional enrichment or relationships among cancer DEGs.

Next, we conducted the same DEG analysis within individual tumor types to explore the potential for tissue-dependent processes involved in ICB response. For melanoma, we only identified 22 cancer DEGs shared between the two melanoma cohorts, and these genes were not enriched in specific pathways. In gastric cancer, we identified 190 cancer ICB response DEGs across the gastric ICB cohort and TCGA cohort. Interestingly, genes associated with poor ICB response were enriched for angiogenesis-related functions (Fig. 4C), with proangiogenic genes ENG, F2R, and PDGFRB overexpressed in ICB nonresponders (MSI/EBV) compared with responders (MSS; Fig. 4D). These data are consistent with the understanding that angiogenesis can be associated with immune suppression (72), suggesting that a proangiogenic environment may impede ICB response in gastric cancer. In urothelial cancer, we found that cancer DEGs were enriched for neuron-related functional terms (Fig. 4E), with neuronal genes overexpressed in ICB responders compared with ICB nonresponders (Fig. 4F). This observation is concordant with a previous study linking the neuronal expression subtype (73) of urothelial cancer with poor overall survival but high response rate to ICB (36).

Overall, while these results indicate an overall paucity of tissue-agnostic, cancer cell–intrinsic features of ICB response on the transcriptomic level, they also highlight the potential for tissue and tumor-type specific expression signatures in cancer cells that may predict ICB response.

### Lack of Cancer-intrinsic Immune Checkpoint Regulation Across Cancer Types

**FIGURE 5:**
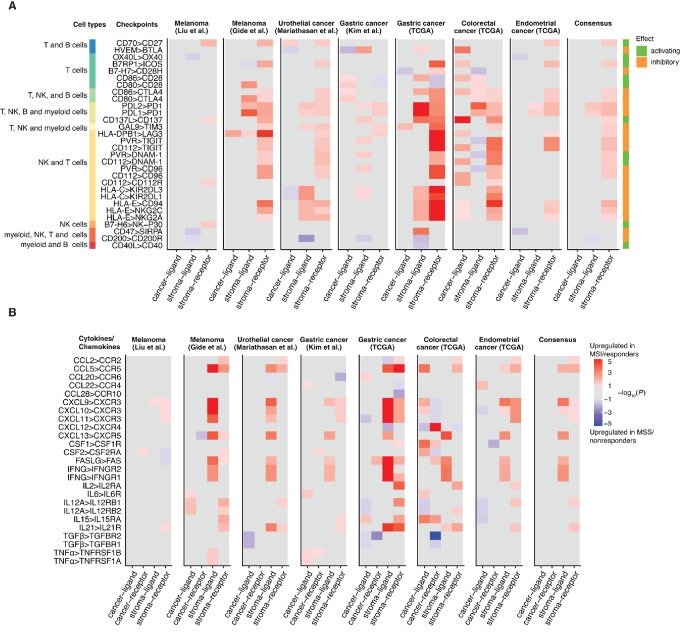
Analysis of ligand and receptor expression of immune modulators. Inferred ligand and receptor expression in the cancer and stroma compartments for immune checkpoint–related proteins (**A**) and cytokines (**B**). Heat maps are colored by the signed −log10(*P*-value) of differential expression between ICB responders and nonresponders (permutation test). Tiles with no differential expression (*P*-value >0.1) were colored gray. In the consensus heat maps, the median signed log_10_(*P*-value) were plotted for genes that were differentially expressed in three out of four ICB cohorts. The immune checkpoint pairs were annotated by the likely immune cell types as well as the effect of the checkpoint interaction (activating or inhibitory).

Although the transcriptome-wide analysis failed to identify conserved cancer-specific DEGs of ICB response, we further explored the expression of immune checkpoint ligands and receptors in cancer and immune cells. We examined the expression patterns of 30 known immune checkpoint ligand-receptor pairs in both the cancer and stromal components of the TME (Materials and Methods), to identify putative ligand-receptor pairs with coordinated expression changes between ICB responders and nonresponders. We found stromal overexpression of 13 checkpoint receptors and two checkpoint ligands to be associated with ICB response across cancer types (Fig. 5A). Checkpoint ligands PD-L1 and PD-L2 and their receptor PD-1 were coordinately upregulated in the stroma of ICB responders compared with ICB nonresponders. PD-L1 (*CD274*) and PD-1 were significantly upregulated in the stroma of ICB responders in six out of seven cohorts studied (*P*-value <0.05, permutation test), while PD-L2 was upregulated in five out of the seven cohorts (Supplementary Fig. S9). Interestingly, no checkpoint ligands were consistently differentially expressed and associated with ICB response in cancer cells across tumor types.

In addition to immune checkpoints, cytokines also play important roles in tumor immunity (74–76). We examined the expression of 13 pairs of cytokines and cytokine receptors known to be involved in tumor immunity (74, 76), aiming to identify cytokine signals associated with ICB response (Fig. 5B). Similar to our analysis for immune checkpoints, we found that cancer-intrinsic cytokine expression was not associated with ICB response across tumor types. However, six cytokines (*IFNG*, *FASLG*, *CXCL13*, *CCL5*, *CXCL10*, and *CXCL9*), and the corresponding receptors for three of the cytokines (*CCR5* for *CCL5*, *CXCR3* for *CXCL9* and *CXCL10*) were significantly upregulated in the stroma of ICB responders, consistent with previous observations (11, 26). Overall, our analysis finds no evidence for a universal mechanism by which cancer cells inhibit immune cells via checkpoints interactions or chemokine/cytokine signaling.

### A Multivariate Classifier Predicts ICB Response Across Cancer Types

**FIGURE 6:**
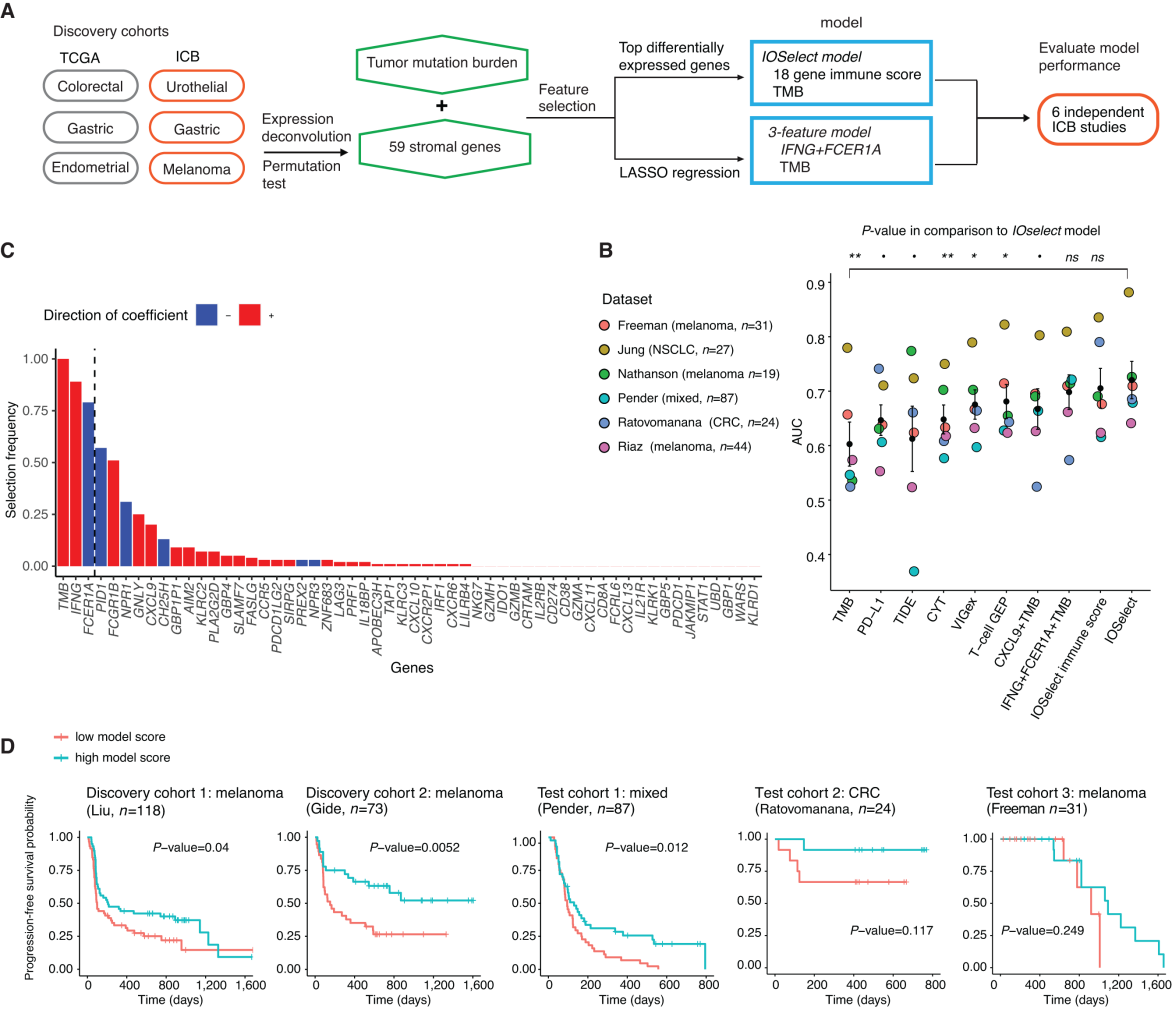
Predictive modeling of response to immune checkpoint inhibition. **A,** Schematic of data and methods used in model training and testing. **B,** Top ranked features of ICB response selected by LASSO logistic regression. **C,** Comparisons of the performance of the *IOselect* model and the 3-feature model (TMB+*IFNG*+*FCER1A*) with existing biomarkers in six independent test cohorts. The differences between the mean AUCs of *IOSelect* and other models were computed using a paired *t* test. **, *P*-value <0.01; *, *P*-value <0.05; *P*-value <0.1. **D,** Kaplan–Meier curves of PFS for patients with high versus low predicted scores (greater than or less than median); *P*-values from log-rank test shown.

Next, we explored whether we could build an accurate predictor of ICB response based on the consensus DEGs identified earlier (Fig. 6A). We focused on the 59 stroma-specific DEGs (Fig. 2A) as they were more consistent across tumor types, in comparison with cancer DEGs, and therefore more likely to be generalize to unseen cancer types. To capture the balance of immune-activating and suppressive signals in the TME, we developed the *IOSelect* immune score, capturing the relative expression difference of response and resistance-associated DEGs (Materials and Methods). While a stromal transcriptomic signature reflects the state and favorability of the TME, TMB is a proxy of the tumor immunogenicity. Because the stromal DEGs and TMB likely represent different facets of tumor immunity, we hypothesized that these could be orthogonal predictors of ICB response. Therefore, we built a multivariate classifier, *IOSelect*, using both the *IOSelect* immune score and TMB as input features. The *IOSelect* classifier was trained on 461 samples from the ICB discovery cohorts with both transcriptomic and genomic data. We evaluated the model performance on six independent test cohorts treated with ICB (28–33), comprising 232 patients with melanoma, colorectal, lung, and other cancers (Materials and Methods). We calculated the area under the receiver operating curve (AUC) of the model, and benchmarked it against established biomarkers of ICB response, namely CXCL9+TMB (16), T-cell GEP (11), VIGex (51), cytolytic score (50), TIDE (13, 49), TMB alone (2, 8), and PD-L1 expression (3). The *IOSelect* classifier achieved a mean AUC of 0.72, significantly outperforming existing biomarkers with mean AUCs ranging from 0.60 (TMB alone) to 0.68 (T-cell GEP; Fig. 6B).

Next, we evaluated whether a simpler model comprising just the top few predictive features could be sufficient to robustly predict ICB response. We used LASSO logistic regression with 10-fold cross-validation to select the most robust predictors from the set of 59 stromal DEGs and TMB (Fig. 6A). We found TMB, *IFNG* expression, and *FCER1A* expression to be the most frequently selected features from 100 bootstrap trials (Fig. 6C). The compact 3-feature model attained a mean AUC of 0.70 across the six test cohorts, which is only marginally lower than the AUC attained by the full *IOSelect* model (Fig. 6B, *P*-value = 0.40 by paired *t* test). Furthermore, the 3-feature classifier is predictive of progression-free survival (PFS) in cohorts with survival data available (Fig. 6D) and to a lesser extent, overall survival (Supplementary Fig. S10). High predicted score is significantly associated with longer PFS in the Gide (27), Liu (26), and Pender (29) cohorts, and is positively associated with PFS in the Ratovomanana (33) and Freeman (30) cohorts although statistical significance is not reached, likely due to the smaller sample sizes of these two cohorts (Fig. 6D). Overall, these results demonstrate that the novel negative biomarkers of ICB response identified from our transcriptome-wide analysis contributes additional predictive power over existing biomarkers.

## Discussion

**FIGURE 7:**
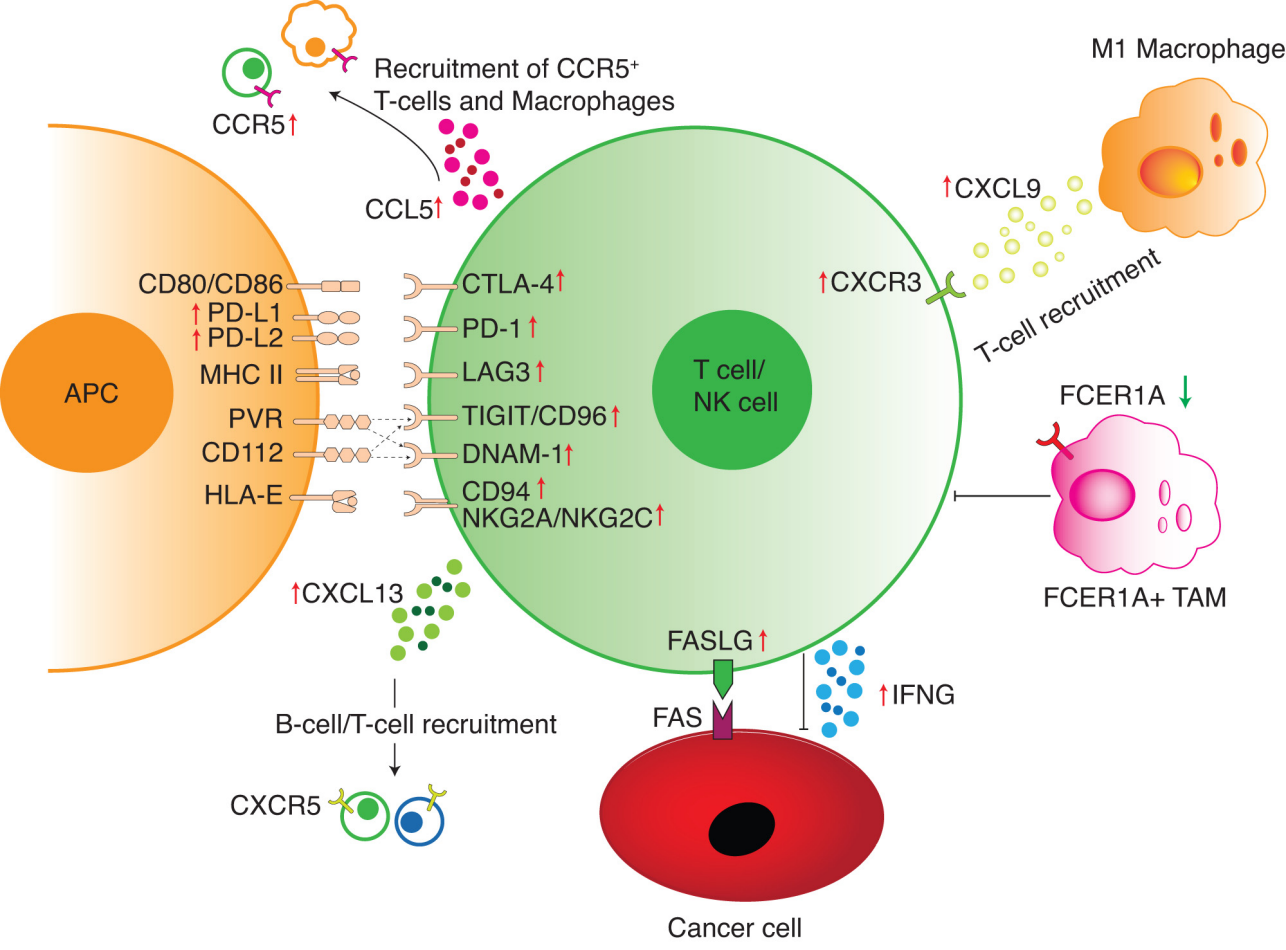
Schematic summarizing the key components associated with ICB response. Red arrows indicate gene positively correlated with ICB response. Green arrow indicates gene negatively correlated with ICB response.

In this study, we performed a transcriptome-wide meta-analysis of ICB response in 1,486 tumors. These tumor samples included 534 tumors with annotated clinical response to ICB treatment, and 952 tumors from cancer types enriched for MSI and ICB-responding tumors. Using *in silico* tumor transcriptome deconvolution, we analyzed gene expression signatures of cancer and their neighboring stromal (nonmalignant) cells and identified DEGs associated with ICB response across tumor types. Strikingly, this meta-analysis revealed a paucity of ICB-response DEGs in cancer cells as compared with stromal cells. The few cancer DEGs that we identified by the meta-analysis were more likely related to the biology of MSI tumors and their DNA mismatch repair deficiency rather than being general determinants of ICB response. In contrast, our analysis revealed 59 stroma-specific ICB-response DEGs, which were strongly enriched in immune-related pathways and processes (Fig. 7).

The two chemokine ligands, *CXCL9* and *CXCL13*, constituted the top two stromal genes overexpressed in ICB responders. Underlining the validity of our approach, these two genes have been highlighted for their roles in antitumor immunity and association with favorable ICB response in multiple recent studies (16, 58, 59). Previous studies suggest CXCL13 may play dual roles in ICB response. First, CXCL13 is produced by Tfh cells and involved in B-cell organization in TLS (56), and the presence of TLS can be a positive predictor of ICB response (57, 69). Second, *CXCL13* has been reported to be highly expressed by neoantigen-reactive CD8^+^ T cells directly involved in cancer cell killing (77, 78). In line with these findings, we found enrichment of both Tfh and CD8^+^ T cells in tumors of ICB responders compared with nonresponders across cancer types (Figs. 3 and 7). In addition, the abundance of these two cell types correlates with *CXCL13* expression. In agreement with recent work, we found that *CXCL9* expression is strongly correlated with M1 macrophage infiltration (58, 79), and M1 macrophages were one of the most differentially enriched immune cell types in ICB responders (Fig. 3A). Although myeloid infiltration has traditionally been associated with immune suppression (65), our data are consistent with the hypothesis that M1 macrophage infiltration, and macrophage polarization, could be an important determinant of tumor sensitivity to ICB.

Intriguingly, we also identified a set of immune-related genes that were negatively associated with ICB response. Among these genes, *FCER1A* and *CH25H* have previously been reported to be overexpressed in anti-inflammatory M2 macrophages (61, 80), and FCER1A+ TAMs have been reported to promote tumor progression by engaging in a positive feedback loop with tumor-initiating cells (62).

Interestingly, we observed an enrichment of the resting states of CD4 memory T cells, dendritic cells, and mast cells in ICB nonresponders. In contrast, the activated states of these cell types are enriched in the tumors of ICB responders (Fig. 3B). Furthermore, while *FCER1A* is expressed in antigen-presenting cells like dendritic cells and macrophages, we noted that its expression is 25 times higher in resting dendritic cells compared to activated dendritic cells, and 14 times higher in M2 macrophages compared with M1 macrophages (52). These results suggest that a preexisting immunosuppressive microenvironment, associated with resting immune cells and high *FCER1A* expression, may prevent effective antitumor immune responses in ICB nonresponders.

Analyses of immune checkpoint and cytokine ligand–receptor interactions revealed coordinated upregulation of several ligand-receptor pairs in the stroma of tumors from ICB responders, including genes encoding PD-L1/PD-L2 and their receptor PD-1, CXCL9/CXCL10 and their receptor CXCR3, and CCL5 and its receptor CCR5 (Figs. 5 and 7). In contrast, we did not find consistent expression changes of checkpoint proteins and cytokines in cancer cells of ICB responders. Among chemokines upregulated in ICB responders, CXCL9, CXCL10, and CXCL13 promote lymphocyte recruitment, while CCL5 is a chemoattractant of both T cells and myeloid cells. CCL5 also directs the recruitment and differentiation of CCR5+ blood monocytes and neutrophils into immunosuppressive TAMs and neutrophils (74, 75). In addition, CCL5 has direct protumoral effects on cancer cells, promoting cancer proliferation and metastasis (74, 75). Preclinical and clinical studies suggested that targeting the CCL5-CCR5 axis could enhance antitumoral response (81, 82). Furthermore, CCR5 was found to be highly expressed by neoantigen-reactive T cells, and overexpressed in ICB responders (16). Here we found both *CCL5* and *CCR5* to be overexpressed in ICB responders, suggesting that targeting CCL5-CCR5 signaling in conjunction with ICB might further potentiate antitumoral immune response. Indeed, a recent trial found modest clinical benefit of anti-CCR5 and anti-PD-1 combination on patient with heavily pretreated microsatellite stable colorectal cancer, and more trials are underway to evaluate the safety and efficacy of anti-CCR5 and checkpoint inhibitor combinations (83). Currently, the majority of companion diagnostics for PD-1/PD-L1 inhibitors measure PD-L1 expression level on either cancer cells alone, or on both cancer cells and infiltrating immune cells, but seldomly on immune cells only (18, 84). Recent clinical trials found PD-L1 expression on immune cells to be prognostic in patients with head and neck cancer (85), non–small cell lung cancer (86), triple-negative breast cancer (87), and renal cell carcinoma (88) treated with ICB. These data are consistent with our results, suggesting that PD-L1 expression on immune cells could be a more consistent marker of ICB response across tumor types. Using the stromal consensus DEGs identified from the meta-analysis, we developed the *IOSelect* score, which robustly predicted ICB response in six independent test cohorts, outperforming existing gene expression signatures for ICB response. Furthermore, we proposed a compact 3-feature model that can attain comparable performance with a single positive marker of immune infiltration (*IFNG*), a single negative marker of immune suppression (*FCER1A*), in combination with TMB. While ideal pan-cancer biomarkers of ICB response should be robust across patient cohorts, we noticed that the Liu and colleagues melanoma cohort (26) did not share many DEGs with the other cohorts, including other melanoma cohorts. This result highlights significant heterogeneity between ICB cohorts, even among cohorts of the same tumor type. It is unclear to what extent this heterogeneity arise from patient selection, differences in ICB regime, differences in prior treatments, different sample collection protocols, or differences in gene expression assays. This highlights the need for large, consistently selected patient cohorts, with standardized gene expression profiling to facilitate systematic biomarker discovery. Furthermore, combining unbiased transcriptome-wide biomarker discovery with prior knowledge-guided analysis of gene signatures may further improve patient selection for ICB.

As cancer cells are under negative selective pressure from the immune system, several mechanisms of immune escape by cancer cells have been proposed: (i) cancer cells downregulate antigen presentation by deleting HLA alleles (71), (ii) cancer cells deplete neoantigens to avoid detection (89), (iii) oncogenic signaling may upregulate PD-L1 expression and suppress T-cell recruitment (90). However, the prevalence of these immune evasion mechanisms are still unclear. A recent meta-analysis found no association between LOH of HLA alleles and ICB response across ICB cohorts (16). Furthermore, a pan-cancer analysis failed to detect neoantigen depletion signals in most tumor types (91). Similarly, we found a lack of conserved cancer-intrinsic mechanisms of immune evasion on the transcriptomic level, which challenge the prevailing dogma that cancer cell–intrinsic expression of checkpoint ligands can modulate immune activity and predict ICB response across cancer types. These insights have profound implications on biomarker assays and *in vitro* model systems for development of improved ICB drugs. These results suggest that cancer cells adopt diverse mechanisms of immune evasion, with no universal strategy adopted by cancer cells to avoid immune-mediated killing. The growing number of molecularly characterized ICB cohorts will enable further exploration of the importance and prevalence of the different cancer-intrinsic immune evasion strategies in determining ICB response.

## Supplementary Material

Supplementary Figure 1 Schematic of bulk tumor expression deconvolution and permutation test to identify differentially expressed genes between responders and non-responders in both cancer and stroma compartments.

Supplementary Figure 2 Validation of bulk tumor expression deconvolution in ICB-treated cohorts.

Supplementary Figure 3 Identification of genes that are differentially expressed between responders and non-responders consistently across discovery cohorts.

Supplementary Figure 4 Evaluation of cell-type specific expression of top DEGs using single cell RNAseq data.

Supplementary Figure 5 Expression of top differentially expressed genes in immune cell subtypes.

Supplementary Figure 6 Differentially enriched immune cell types between responders and non-responders.

Supplementary Figure 7 Correlations between the abundance of immune cell subtypes with the gene expression of top stromal biomarkers in individual cohorts.

Supplementary Figure 8 Area under the receiver operating curve (AUC) of the cancer-DEG model on 6 test datasets.

Supplementary Figure 9 Supplementary Figure 9

Supplementary Figure 10 Association between model prediction and overall survival.

Supplementary Table 1 Clinical characteristics of the study cohorts.

Supplementary Table 2 Mean correlation coefficients between stroma DES and immune cell subsets across all discovery cohorts.

Supplementary Table 3 Data source of the ICB cohort.

## References

[1] O'Reilly EM , Oh D-Y, Dhani N, Renouf DJ, Lee MA, Sun W, . Durvalumab with or without tremelimumab for patients with metastatic pancreatic ductal adenocarcinoma: a phase 2 randomized clinical trial. JAMA Oncol 2019;5:1431–8. 31318392 10.1001/jamaoncol.2019.1588 PMC6647002

[2] Yarchoan M , Hopkins A, Jaffee EM. Tumor mutational burden and response rate to PD-1 inhibition. N Engl J Med 2017;377:2500–1. 29262275 10.1056/NEJMc1713444 PMC6549688

[3] Havel JJ , Chowell D, Chan TA. The evolving landscape of biomarkers for checkpoint inhibitor immunotherapy. Nat Rev Cancer 2019;19:133–50. 30755690 10.1038/s41568-019-0116-x PMC6705396

[4] Marcus L , Lemery SJ, Keegan P, Pazdur R. FDA approval summary: pembrolizumab for the treatment of microsatellite instability-high solid tumors. Clin Cancer Res 2019;25:3753–8. 30787022 10.1158/1078-0432.CCR-18-4070

[5] FDA. FDA approves pembrolizumab for adults and children with TMB-H solid tumors; 2020.

[6] Banchereau R , Leng N, Zill O, Sokol E, Liu G, Pavlick D, . Molecular determinants of response to PD-L1 blockade across tumor types. Nat Commun 2021;12:3969. 34172722 10.1038/s41467-021-24112-w PMC8233428

[7] Valero C , Lee M, Hoen D, Zehir A, Berger MF, Seshan VE, . Response rates to anti-PD-1 immunotherapy in microsatellite-stable solid tumors with 10 or more mutations per megabase. JAMA Oncol 2021;7:739–43. 33599686 10.1001/jamaoncol.2020.7684 PMC7893543

[8] Samstein RM , Lee C-H, Shoushtari AN, Hellmann MD, Shen R, Janjigian YY, . Tumor mutational load predicts survival after immunotherapy across multiple cancer types. Nat Genet 2019;51:202–6. 30643254 10.1038/s41588-018-0312-8 PMC6365097

[9] Cortes-Ciriano I , Lee S, Park WY, Kim TM, Park PJ. A molecular portrait of microsatellite instability across multiple cancers. Nat Commun 2017;8:15180. 28585546 10.1038/ncomms15180 PMC5467167

[10] Bonneville R , Krook MA, Kautto EA, Miya J, Wing MR, Chen H-Z, . Landscape of microsatellite instability across 39 cancer types. JCO Precis Oncol 2017;2017:PO.17.00073. 29850653 10.1200/PO.17.00073 PMC5972025

[11] Ayers M , Lunceford J, Nebozhyn M, Murphy E, Loboda A, Kaufman DR, . IFN-γ-related mRNA profile predicts clinical response to PD-1 blockade. J Clin Invest 2017;127:2930–40. 28650338 10.1172/JCI91190 PMC5531419

[12] Cristescu R , Mogg R, Ayers M, Albright A, Murphy E, Yearley J, . Pan-tumor genomic biomarkers for PD-1 checkpoint blockade-based immunotherapy. Science 2018;362:eaar3593. 30309915 10.1126/science.aar3593 PMC6718162

[13] Jiang P , Gu S, Pan D, Fu J, Sahu A, Hu X, . Signatures of T cell dysfunction and exclusion predict cancer immunotherapy response. Nat Med 2018;24:1550–8. 30127393 10.1038/s41591-018-0136-1 PMC6487502

[14] Bagaev A , Kotlov N, Nomie K, Svekolkin V, Gafurov A, Isaeva O, . Conserved pan-cancer microenvironment subtypes predict response to immunotherapy. Cancer Cell 2021;39:845–65. 34019806 10.1016/j.ccell.2021.04.014

[15] Subramanian M , Kabir AU, Barisas D, Krchma K, Choi K. Conserved angio-immune subtypes of the tumor microenvironment predict response to immune checkpoint blockade therapy. Cell Rep Med 2023;4:100896. 36630952 10.1016/j.xcrm.2022.100896 PMC9873950

[16] Litchfield K , Reading JL, Puttick C, Thakkar K, Abbosh C, Bentham R, . Meta-analysis of tumor- and T cell-intrinsic mechanisms of sensitization to checkpoint inhibition. Cell 2021;184:596–14. 33508232 10.1016/j.cell.2021.01.002 PMC7933824

[17] Bareche Y , Kelly D, Abbas-Aghababazadeh F, Nakano M, Esfahani PN, Tkachuk D, . Leveraging big data of immune checkpoint blockade response identifies novel potential targets. Ann Oncol 2022;33:1304–17. 36055464 10.1016/j.annonc.2022.08.084

[18] Nishino M , Ramaiya NH, Hatabu H, Hodi FS. Monitoring immune-checkpoint blockade: response evaluation and biomarker development. Nat Rev Clin Oncol 2017;14:655–68.28653677

[19] Guruprasad P , Lee YG, Kim KH, Ruella M. The current landscape of single-cell transcriptomics for cancer immunotherapy. J Exp Med 2021;218:e20201574. 33601414 10.1084/jem.20201574 PMC7754680

[20] Gohil SH , Iorgulescu JB, Braun DA, Keskin DB, Livak KJ. Applying high-dimensional single-cell technologies to the analysis of cancer immunotherapy. Nat Rev Clin Oncol 2021;18:244–56. 33277626 10.1038/s41571-020-00449-x PMC8415132

[21] Zhong Y , Liu Z. Gene expression deconvolution in linear space. Nat Methods 2011;9:8–9. 22205510 10.1038/nmeth.1830

[22] Ghoshdastider U , Rohatgi N, Mojtabavi Naeini M, Baruah P, Revkov E, Guo YA, . Pan-cancer analysis of ligand-receptor cross-talk in the tumor microenvironment. Cancer Res 2021;81:1802–12. 33547160 10.1158/0008-5472.CAN-20-2352

[23] Rohatgi N , Ghoshdastider U, Baruah P, Kulshrestha T, Skanderup AJ. A pan-cancer metabolic atlas of the tumor microenvironment. Cell Rep. 2022;39:110800. 35545044 10.1016/j.celrep.2022.110800

[24] Kim ST , Cristescu R, Bass AJ, Kim K-M, Odegaard JI, Kim K, . Comprehensive molecular characterization of clinical responses to PD-1 inhibition in metastatic gastric cancer. Nat Med 2018;24:1449–58. 30013197 10.1038/s41591-018-0101-z

[25] Mariathasan S , Turley SJ, Nickles D, Castiglioni A, Yuen K, Wang Y, . TGFβ attenuates tumour response to PD-L1 blockade by contributing to exclusion of T cells. Nature 2018;554:544–8. 29443960 10.1038/nature25501 PMC6028240

[26] Liu D , Schilling B, Liu D, Sucker A, Livingstone E, Jerby-Arnon L, . Integrative molecular and clinical modeling of clinical outcomes to PD1 blockade in patients with metastatic melanoma. Nat Med 2019;25:1916–27. 31792460 10.1038/s41591-019-0654-5 PMC6898788

[27] Gide TN , Quek C, Menzies AM, Tasker AT, Shang P, Holst J, . Distinct immune cell populations define response to anti-PD-1 monotherapy and anti-PD-1/anti-CTLA-4 combined therapy. Cancer Cell 2019;35:238–55. 30753825 10.1016/j.ccell.2019.01.003

[28] Riaz N , Havel JJ, Makarov V, Desrichard A, Urba WJ, Sims JS, . Tumor and microenvironment evolution during immunotherapy with nivolumab. Cell 2017;171:934–49. 29033130 10.1016/j.cell.2017.09.028 PMC5685550

[29] Pender A , Titmuss E, Pleasance ED, Fan KY, Pearson H, Brown SD, . Genome and transcriptome biomarkers of response to immune checkpoint inhibitors in advanced solid tumors. Clin Cancer Res 2021;27:202–12. 33020056 10.1158/1078-0432.CCR-20-1163

[30] Freeman SS , Sade-Feldman M, Kim J, Stewart C, Gonye ALK, Ravi A, . Combined tumor and immune signals from genomes or transcriptomes predict outcomes of checkpoint inhibition in melanoma. Cell Rep Med 2022;3:100500. 35243413 10.1016/j.xcrm.2021.100500 PMC8861826

[31] Jung H , Kim HS, Kim JY, Sun J-M, Ahn JS, Ahn M-J, . DNA methylation loss promotes immune evasion of tumours with high mutation and copy number load. Nat Commun 2019;10:4278. 31537801 10.1038/s41467-019-12159-9 PMC6753140

[32] Nathanson T , Ahuja A, Rubinsteyn A, Aksoy BA, Hellmann MD, Miao D, . Somatic mutations and neoepitope homology in melanomas treated with CTLA-4 blockade. Cancer Immunol Res 2017;5:84–91. 27956380 10.1158/2326-6066.CIR-16-0019 PMC5253347

[33] Ratovomanana T , Nicolle R, Cohen R, Diehl A, Siret A, Letourneur Q, . Prediction of response to immune checkpoint blockade in patients with metastatic colorectal cancer with microsatellite instability. Ann Oncol 2023;34:703–13. 37269904 10.1016/j.annonc.2023.05.010

[34] Dobin A , Davis CA, Schlesinger F, Drenkow J, Zaleski C, Jha S, . STAR: ultrafast universal RNA-seq aligner. Bioinformatics 2013;29:15–21. 23104886 10.1093/bioinformatics/bts635 PMC3530905

[35] Patro R , Duggal G, Love MI, Irizarry RA, Kingsford C. Salmon provides fast and bias-aware quantification of transcript expression. Nat Methods 2017;14:417–9. 28263959 10.1038/nmeth.4197 PMC5600148

[36] Kim J , Kwiatkowski D, McConkey DJ, Meeks JJ, Freeman SS, Bellmunt J, . The Cancer Genome Atlas expression subtypes stratify response to checkpoint inhibition in advanced urothelial cancer and identify a subset of patients with high survival probability. Eur Urol 2019;75:961–4. 30851984 10.1016/j.eururo.2019.02.017

[37] Revkov E , Kulshrestha T, Sung KW, Skanderup AJ. PUREE: accurate pan-cancer tumor purity estimation from gene expression data. Commun Biol 2023;6:394. 37041233 10.1038/s42003-023-04764-8 PMC10090153

[38] Yoshihara K , Shahmoradgoli M, Martínez E, Vegesna R, Kim H, Torres-Garcia W, . Inferring tumour purity and stromal and immune cell admixture from expression data. Nat Commun 2013;4:2612. 24113773 10.1038/ncomms3612 PMC3826632

[39] Favero F , Joshi T, Marquard AM, Birkbak NJ, Krzystanek M, Li Q, . Sequenza: allele-specific copy number and mutation profiles from tumor sequencing data. Ann Oncol 2015;26:64–70. 25319062 10.1093/annonc/mdu479 PMC4269342

[40] Larson NB , Fridley BL. PurBayes: estimating tumor cellularity and subclonality in next-generation sequencing data. Bioinformatics 2013;29:1888–9. 23749958 10.1093/bioinformatics/btt293 PMC3712213

[41] Liu Y , Sethi NS, Hinoue T, Schneider BG, Cherniack AD, Sanchez-Vega F, . Comparative molecular analysis of gastrointestinal adenocarcinomas. Cancer Cell 2018;33:721–35. 29622466 10.1016/j.ccell.2018.03.010 PMC5966039

[42] Bindea G , Mlecnik B, Hackl H, Charoentong P, Tosolini M, Kirilovsky A, . ClueGO: a cytoscape plug-in to decipher functionally grouped gene ontology and pathway annotation networks. Bioinformatics 2009;25:1091–3. 19237447 10.1093/bioinformatics/btp101 PMC2666812

[43] Bi K , He MX, Bakouny Z, Kanodia A, Napolitano S, Wu J, . Tumor and immune reprogramming during immunotherapy in advanced renal cell carcinoma. Cancer Cell 2021;39:649–61. 33711272 10.1016/j.ccell.2021.02.015 PMC8115394

[44] Koboldt DC , Zhang Q, Larson DE, Shen D, McLellan MD, Lin L, . VarScan 2: somatic mutation and copy number alteration discovery in cancer by exome sequencing. Genome Res 2012;22:568–76. 22300766 10.1101/gr.129684.111 PMC3290792

[45] Cibulskis K , Lawrence MS, Carter SL, Sivachenko A, Jaffe D, Sougnez C, . Sensitive detection of somatic point mutations in impure and heterogeneous cancer samples. Nat Biotechnol 2013;31:213–9. 23396013 10.1038/nbt.2514 PMC3833702

[46] Lai Z , Markovets A, Ahdesmaki M, Chapman B, Hofmann O, McEwen R, . VarDict: a novel and versatile variant caller for next-generation sequencing in cancer research. Nucleic Acids Res 2016;44:e108. 27060149 10.1093/nar/gkw227 PMC4914105

[47] Garrison E , Marth G. Haplotype-based variant detection from short-read sequencing. ArXiv e-prints 2012;1207. Available from: http://adsabs.harvard.edu/abs/2012arXiv1207.3907G.

[48] Huang W , Guo YA, Muthukumar K, Baruah P, Chang MM, Jacobsen Skanderup A. SMuRF: portable and accurate ensemble prediction of somatic mutations. Bioinformatics 2019;35:3157–9. 30649191 10.1093/bioinformatics/btz018 PMC6735703

[49] Fu J , Li K, Zhang W, Wan C, Zhang J, Jiang P, . Large-scale public data reuse to model immunotherapy response and resistance. Genome Med 2020;12:21. 32102694 10.1186/s13073-020-0721-z PMC7045518

[50] Rooney MS , Shukla SA, Wu CJ, Getz G, Hacohen N. Molecular and genetic properties of tumors associated with local immune cytolytic activity. Cell 2015;160:48–61. 25594174 10.1016/j.cell.2014.12.033 PMC4856474

[51] Hernando-Calvo A , Vila-Casadesús M, Bareche Y, Gonzalez-Medina A, Abbas-Aghababazadeh F, Lo Giacco D, . A pan-cancer clinical platform to predict immunotherapy outcomes and prioritize immuno-oncology combinations in early-phase trials. Med 2023;4:710–27. 37572657 10.1016/j.medj.2023.07.006

[52] Newman AM , Steen CB, Liu CL, Gentles AJ, Chaudhuri AA, Scherer F, . Determining cell type abundance and expression from bulk tissues with digital cytometry. Nat Biotechnol 2019;37:773–82. 31061481 10.1038/s41587-019-0114-2 PMC6610714

[53] Green AK , Feinberg J, Makker V. A review of immune checkpoint blockade therapy in endometrial cancer. Am Soc Clin Oncol Educ Book 2020;40:1–7. 10.1200/EDBK_280503 32213091

[54] Kubota Y , Kawazoe A, Sasaki A, Mishima S, Sawada K, Nakamura Y, . The impact of molecular subtype on efficacy of chemotherapy and checkpoint inhibition in advanced gastric cancer. Clin Cancer Res 2020;26:3784–90. 32156744 10.1158/1078-0432.CCR-20-0075

[55] Gorbachev AV , Kobayashi H, Kudo D, Tannenbaum CS, Finke JH, Shu S, . CXC chemokine ligand 9/monokine induced by IFN-gamma production by tumor cells is critical for T cell-mediated suppression of cutaneous tumors. J Immunol 2007;178:2278–86. 17277133 10.4049/jimmunol.178.4.2278

[56] Kazanietz MG , Durando M, Cooke M. CXCL13 and its receptor CXCR5 in cancer: inflammation, immune response, and beyond. Front Endocrinol 2019;10:471. 10.3389/fendo.2019.00471 PMC6639976 31354634

[57] Hollern DP , Xu N, Thennavan A, Glodowski C, Garcia-Recio S, Mott KR, . B cells and T follicular helper cells mediate response to checkpoint inhibitors in high mutation burden mouse models of breast cancer. Cell 2019;179:1191–206. 31730857 10.1016/j.cell.2019.10.028 PMC6911685

[58] Qu Y , Wen J, Thomas G, Yang W, Prior W, He W, . Baseline frequency of inflammatory Cxcl9-expressing tumor-associated macrophages predicts response to avelumab treatment. Cell Rep 2020;32:107873. 32640238 10.1016/j.celrep.2020.107873

[59] Goswami S , Chen Y, Anandhan S, Szabo PM, Basu S, Blando JM, . *ARID1A* mutation plus CXCL13 expression act as combinatorial biomarkers to predict responses to immune checkpoint therapy in mUCC. Sci Transl Med 2020;12:eabc4220. 32554706 10.1126/scitranslmed.abc4220

[60] Fehrenbacher L , Spira A, Ballinger M, Kowanetz M, Vansteenkiste J, Mazieres J, . Atezolizumab versus docetaxel for patients with previously treated non-small-cell lung cancer (POPLAR): a multicentre, open-label, phase 2 randomised controlled trial. Lancet 2016;387:1837–46. 26970723 10.1016/S0140-6736(16)00587-0

[61] Pellizzari G , Hoskin C, Crescioli S, Mele S, Gotovina J, Chiaruttini G, . IgE re-programs alternatively-activated human macrophages towards pro-inflammatory anti-tumoural states. EBioMedicine 2019;43:67–81. 30956175 10.1016/j.ebiom.2019.03.080 PMC6562024

[62] Taniguchi S , Elhance A, Van Duzer A, Kumar S, Leitenberger JJ, Oshimori N. Tumor-initiating cells establish an IL-33-TGF-β niche signaling loop to promote cancer progression. Science 2020;369:eaay1813. 32675345 10.1126/science.aay1813 PMC10870826

[63] Cyster JG , Dang EV, Reboldi A, Yi T. 25-Hydroxycholesterols in innate and adaptive immunity. Nat Rev Immunol 2014;14:731–43. 25324126 10.1038/nri3755

[64] Kerkela R , Ulvila J, Magga J. Natriuretic peptides in the regulation of cardiovascular physiology and metabolic events. J Am Heart Assoc 2015;4:e002423. 26508744 10.1161/JAHA.115.002423 PMC4845118

[65] Duan Z , Luo Y. Targeting macrophages in cancer immunotherapy. Signal Transduct Target Ther 2021;6:127. 33767177 10.1038/s41392-021-00506-6 PMC7994399

[66] Zeng D , Ye Z, Wu J, Zhou R, Fan X, Wang G, . Macrophage correlates with immunophenotype and predicts anti-PD-L1 response of urothelial cancer. Theranostics 2020;10:7002–14. 32550918 10.7150/thno.46176 PMC7295060

[67] Crotty S . T follicular helper cell biology: a decade of discovery and diseases. Immunity 2019;50:1132–48. 31117010 10.1016/j.immuni.2019.04.011 PMC6532429

[68] Garaud S , Buisseret L, Solinas C, Gu-Trantien C, de Wind A, Van den Eynden G, . Tumor infiltrating B-cells signal functional humoral immune responses in breast cancer. JCI Insight 2019;5:e129641. 31408436 10.1172/jci.insight.129641 PMC6795287

[69] Cui C , Wang J, Fagerberg E, Chen P-M, Connolly KA, Damo M, . Neoantigen-driven B cell and CD4 T follicular helper cell collaboration promotes anti-tumor CD8 T cell responses. Cell 2021;184:6101–18. 34852236 10.1016/j.cell.2021.11.007 PMC8671355

[70] Bindea G , Mlecnik B, Tosolini M, Kirilovsky A, Waldner M, Obenauf AC, . Spatiotemporal dynamics of intratumoral immune cells reveal the immune landscape in human cancer. Immunity 2013;39:782–95. 24138885 10.1016/j.immuni.2013.10.003

[71] Kalbasi A , Ribas A. Tumour-intrinsic resistance to immune checkpoint blockade. Nat Rev Immunol 2020;20:25–39. 31570880 10.1038/s41577-019-0218-4 PMC8499690

[72] Motz GT , Coukos G. The parallel lives of angiogenesis and immunosuppression: cancer and other tales. Nat Rev Immunol 2011;11:702–11. 21941296 10.1038/nri3064

[73] Robertson AG , Kim J, Al-Ahmadie H, Bellmunt J, Guo G, Cherniack AD, . Comprehensive molecular characterization of muscle-invasive bladder cancer. Cell 2018;174:1033. 30096301 10.1016/j.cell.2018.07.036 PMC6297116

[74] Nagarsheth N , Wicha MS, Zou W. Chemokines in the cancer microenvironment and their relevance in cancer immunotherapy. Nat Rev Immunol 2017;17:559–72. 28555670 10.1038/nri.2017.49 PMC5731833

[75] Mollica Poeta V , Massara M, Capucetti A, Bonecchi R. Chemokines and chemokine receptors: new targets for cancer immunotherapy. Front Immunol 2019;10:379. 30894861 10.3389/fimmu.2019.00379 PMC6414456

[76] Dranoff G . Cytokines in cancer pathogenesis and cancer therapy. Nat Rev Cancer 2004;4:11–22. 14708024 10.1038/nrc1252

[77] Zheng C , Fass JN, Shih Y-P, Gunderson AJ, Sanjuan Silva N, Huang H, . Transcriptomic profiles of neoantigen-reactive T cells in human gastrointestinal cancers. Cancer Cell 2022;40:410–23. 35413272 10.1016/j.ccell.2022.03.005

[78] Lowery FJ , Krishna S, Yossef R, Parikh NB, Chatani PD, Zacharakis N, . Molecular signatures of antitumor neoantigen-reactive T cells from metastatic human cancers. Science 2022;375:877–84. 35113651 10.1126/science.abl5447 PMC8996692

[79] House IG , Savas P, Lai J, Chen AXY, Oliver AJ, Teo ZL, . Macrophage-derived CXCL9 and CXCL10 are required for antitumor immune responses following immune checkpoint blockade. Clin Cancer Res 2020;26:487–504. 31636098 10.1158/1078-0432.CCR-19-1868

[80] Preuss I , Ludwig M-G, Baumgarten B, Bassilana F, Gessier F, Seuwen K, . Transcriptional regulation and functional characterization of the oxysterol/EBI2 system in primary human macrophages. Biochem Biophys Res Commun 2014;446:663–8. 24480442 10.1016/j.bbrc.2014.01.069

[81] Halama N , Zoernig I, Berthel A, Kahlert C, Klupp F, Suarez-Carmona M, . Tumoral immune cell exploitation in colorectal cancer metastases can be targeted effectively by anti-CCR5 therapy in cancer patients. Cancer Cell 2016;29:587–601. 27070705 10.1016/j.ccell.2016.03.005

[82] Ban Y , Mai J, Li X, Mitchell-Flack M, Zhang T, Zhang L, . Targeting autocrine CCL5-CCR5 axis reprograms immunosuppressive myeloid cells and reinvigorates antitumor immunity. Cancer Res 2017;77:2857–68. 28416485 10.1158/0008-5472.CAN-16-2913 PMC5484057

[83] Haag GM , Springfeld C, Grün B, Apostolidis L, Zschäbitz S, Dietrich M, . Pembrolizumab and maraviroc in refractory mismatch repair proficient/microsatellite-stable metastatic colorectal cancer – The PICCASSO phase I trial. Eur J Cancer 2022;167:112–22. 35427833 10.1016/j.ejca.2022.03.017

[84] Twomey JD , Zhang B. Cancer immunotherapy update: FDA-approved checkpoint inhibitors and companion diagnostics. AAPS J 2021;23:39. 33677681 10.1208/s12248-021-00574-0 PMC7937597

[85] Blazek T , Petras M, Knybel L, Cvek J, Soumarova R. Programmed cell death ligand 1 expression on immune cells and survival in patients with nonmetastatic head and neck cancer: a systematic review and meta-analysis. JAMA Netw Open 2023;6:e236324. 37000447 10.1001/jamanetworkopen.2023.6324 PMC10066461

[86] Liu Y , Zugazagoitia J, Ahmed FS, Henick BS, Gettinger SN, Herbst RS, . Immune cell PD-L1 colocalizes with macrophages and is associated with outcome in PD-1 pathway blockade therapy. Clin Cancer Res 2020;26:970–7. 31615933 10.1158/1078-0432.CCR-19-1040 PMC7024671

[87] Schmid P , Adams S, Rugo HS, Schneeweiss A, Barrios CH, Iwata H, . Atezolizumab and nab-paclitaxel in advanced triple-negative breast cancer. N Engl J Med 2018;379:2108–21. 30345906 10.1056/NEJMoa1809615

[88] Choueiri TK , Motzer RJ, Rini BI, Haanen J, Campbell MT, Venugopal B, . Updated efficacy results from the JAVELIN Renal 101 trial: first-line avelumab plus axitinib versus sunitinib in patients with advanced renal cell carcinoma. Ann Oncol 2020;31:1030–9. 32339648 10.1016/j.annonc.2020.04.010 PMC8436592

[89] Zapata L , Caravagna G, Williams MJ, Lakatos E, AbdulJabbar K, Werner B, . Immune selection determines tumor antigenicity and influences response to checkpoint inhibitors. Nat Genet 2023;55:451–60. 36894710 10.1038/s41588-023-01313-1 PMC10011129

[90] Spranger S , Gajewski TF. Impact of oncogenic pathways on evasion of antitumour immune responses. Nat Rev Cancer 2018;18:139–47. 29326431 10.1038/nrc.2017.117 PMC6685071

[91] Van den Eynden J , Jimenez-Sanchez A, Miller ML, Larsson E. Lack of detectable neoantigen depletion signals in the untreated cancer genome. Nat Genet 2019;51:1741–8. 31768072 10.1038/s41588-019-0532-6 PMC6887557

