# Supplementary material for: Transcriptome Deconvolution Reveals Absence of Cancer Cell Expression Signature in Immune Checkpoint Blockade Response
Source: Cancer Res Commun. 2024 Jun 26;4(6):1581–96. doi: 10.1158/2767-9764.CRC-23-0442 (PMC11203396; doi:10.1158/2767-9764.CRC-23-0442)
Supplement: Supplementary Figure 1 — Schematic of bulk tumor expression deconvolution and permutation test to identify differentially expressed genes between responders and non-responders in both cancer and stroma compartments. [file crc-23-0442-s01.pdf]

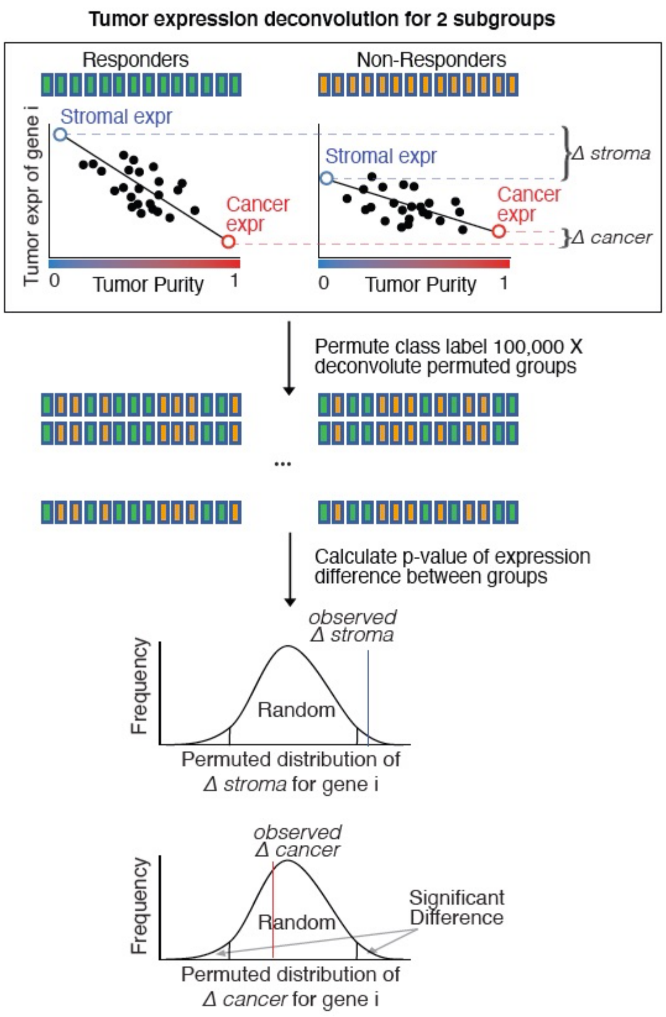

Supplementary Figure 1. **Schematic of bulk tumor expression deconvolution and permutation test to identify differentially expressed genes between responders and non-responders in both cancer and stroma compartments.**
