# Supplementary material for: Transcriptome Deconvolution Reveals Absence of Cancer Cell Expression Signature in Immune Checkpoint Blockade Response
Source: Cancer Res Commun. 2024 Jun 26;4(6):1581–96. doi: 10.1158/2767-9764.CRC-23-0442 (PMC11203396; doi:10.1158/2767-9764.CRC-23-0442)
Supplement: Supplementary Figure 2 — Validation of bulk tumor expression deconvolution in ICB-treated cohorts. [file crc-23-0442-s02.pdf]

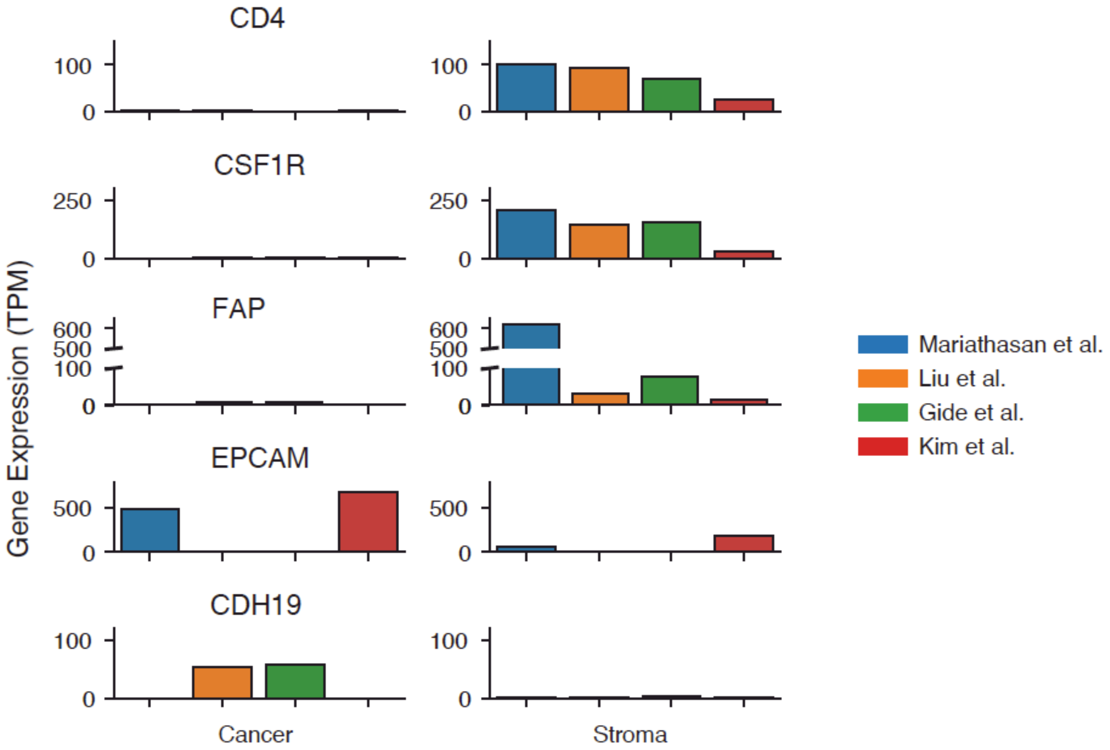

Supplementary Figure 2. Validation of bulk tumor expression deconvolution in ICB-treated cohorts. Inferred expression of known lineage-specific genes in cancer and stroma compartments across ICB cohorts.
