# Supplementary material for: Transcriptome Deconvolution Reveals Absence of Cancer Cell Expression Signature in Immune Checkpoint Blockade Response
Source: Cancer Res Commun. 2024 Jun 26;4(6):1581–96. doi: 10.1158/2767-9764.CRC-23-0442 (PMC11203396; doi:10.1158/2767-9764.CRC-23-0442)
Supplement: Supplementary Figure 3 — Identification of genes that are differentially expressed between responders and non-responders consistently across discovery cohorts. [file crc-23-0442-s03.pdf]

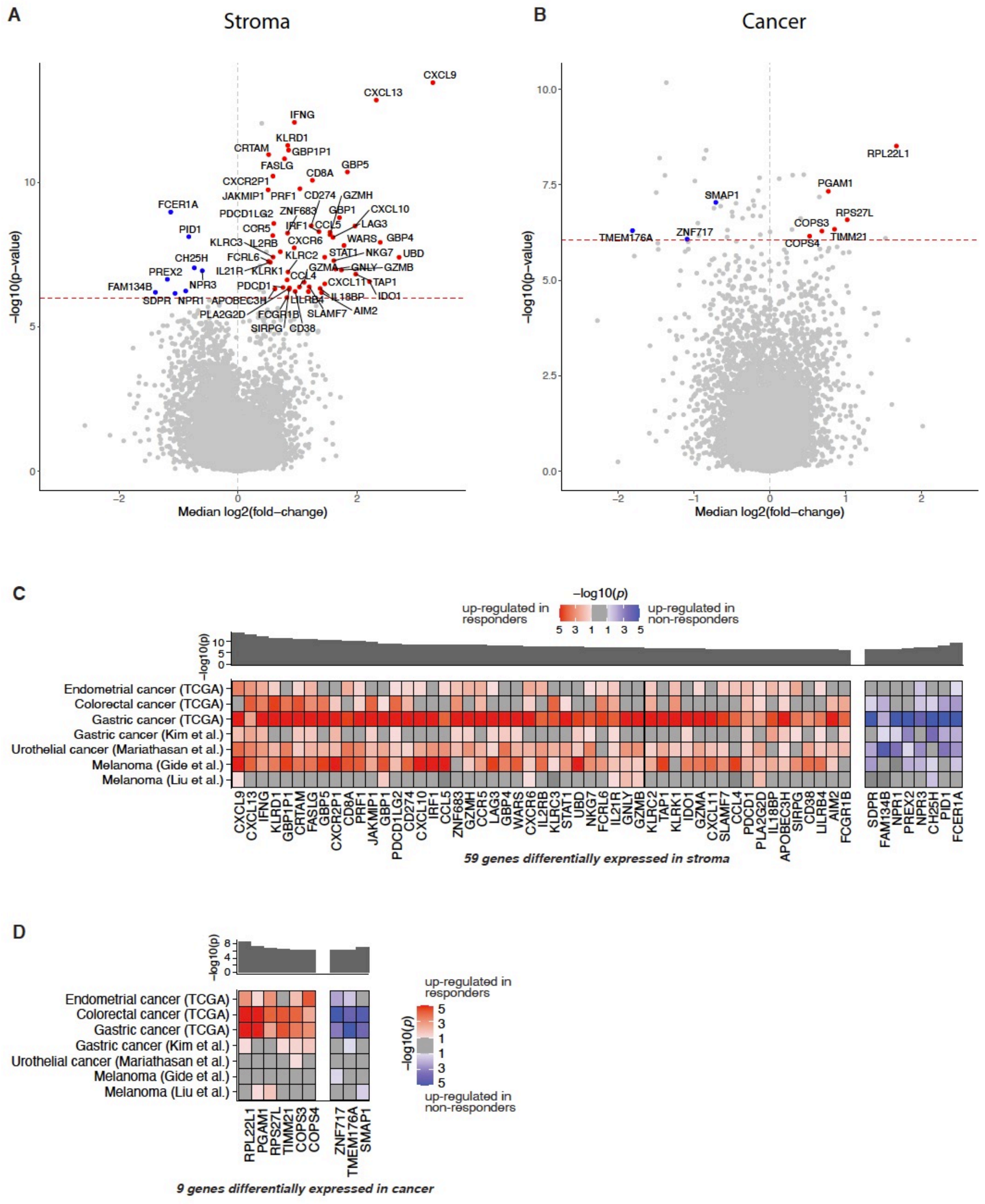

Supplementary Figure 3. **Identification of genes that are differentially expressed between responders and non-responders consistently across discovery cohorts.** Volcano plots of differentially expressed genes in the **(A)** stromal and **(B)** cancer compartments. The y-axis is the -log10(meta p-value) of differential expression. The meta p-value is calculated using the Fisher's method, combining p-values all cohorts. The x-axis is the median log2(fold-change) of gene expression of responders (MSI/EBV) over non-responders (MSS). The red line represents the q-value<0.01 cutoff. Genes with median absolute log2(fold-change)>0.5, q-value <0.01, and p-value<0.1 in at least one ICB cohort are colored and labelled. Heatmaps

of differentially expressed genes in the **(C)** stroma and **(D)** cancer compartments. Heatmaps are colored by the signed -log10(*p*-value) of differentially expression. Genes with median absolute log2(fold-change)>0.5, q-value <0.01, and p-value<0.1 in at least one ICB cohort are shown.
