# Supplementary material for: Transcriptome Deconvolution Reveals Absence of Cancer Cell Expression Signature in Immune Checkpoint Blockade Response
Source: Cancer Res Commun. 2024 Jun 26;4(6):1581–96. doi: 10.1158/2767-9764.CRC-23-0442 (PMC11203396; doi:10.1158/2767-9764.CRC-23-0442)
Supplement: Supplementary Figure 4 — Evaluation of cell-type specific expression of top DEGs using single cell RNAseq data. [file crc-23-0442-s04.pdf]

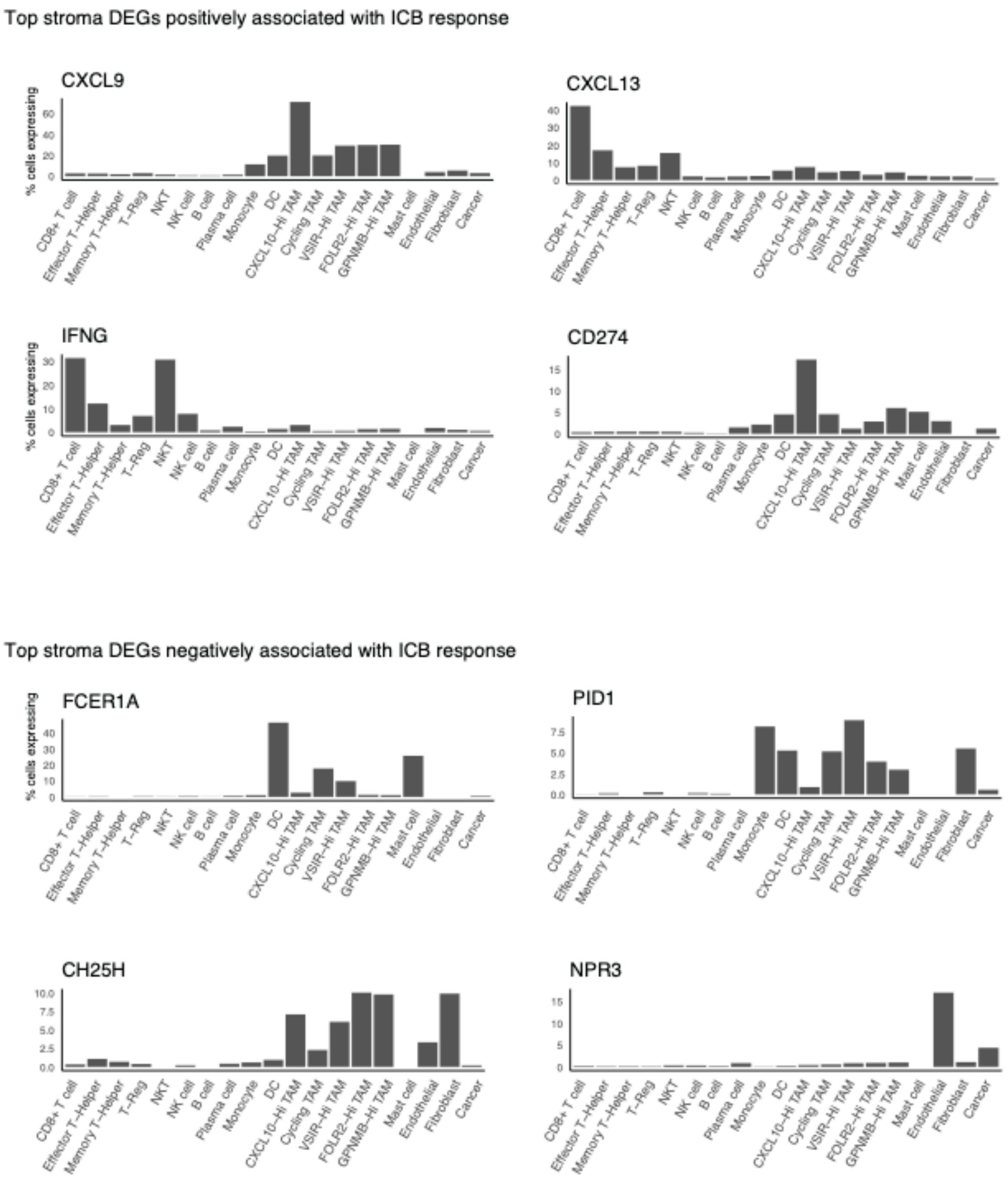

Supplementary Figure 4. Evaluation of cell-type specific expression of top DEGs using single cell RNAseq data). Barplots of the percentage of cells expressing each gene for individual cell types**,** using data from scRNAseq of renal cell cancer patients treated with ICB (Bi. et al., *Cancer Cell*, 2021). CXCL10-Hi TAM are M1-like, while VSIR-Hi, FOLR2-Hi and GPNMB-Hi TAMs are M2-like TAMs.
