# Supplementary material for: Transcriptome Deconvolution Reveals Absence of Cancer Cell Expression Signature in Immune Checkpoint Blockade Response
Source: Cancer Res Commun. 2024 Jun 26;4(6):1581–96. doi: 10.1158/2767-9764.CRC-23-0442 (PMC11203396; doi:10.1158/2767-9764.CRC-23-0442)
Supplement: Supplementary Figure 5 — Expression of top differentially expressed genes in immune cell subtypes. [file crc-23-0442-s05.pdf]

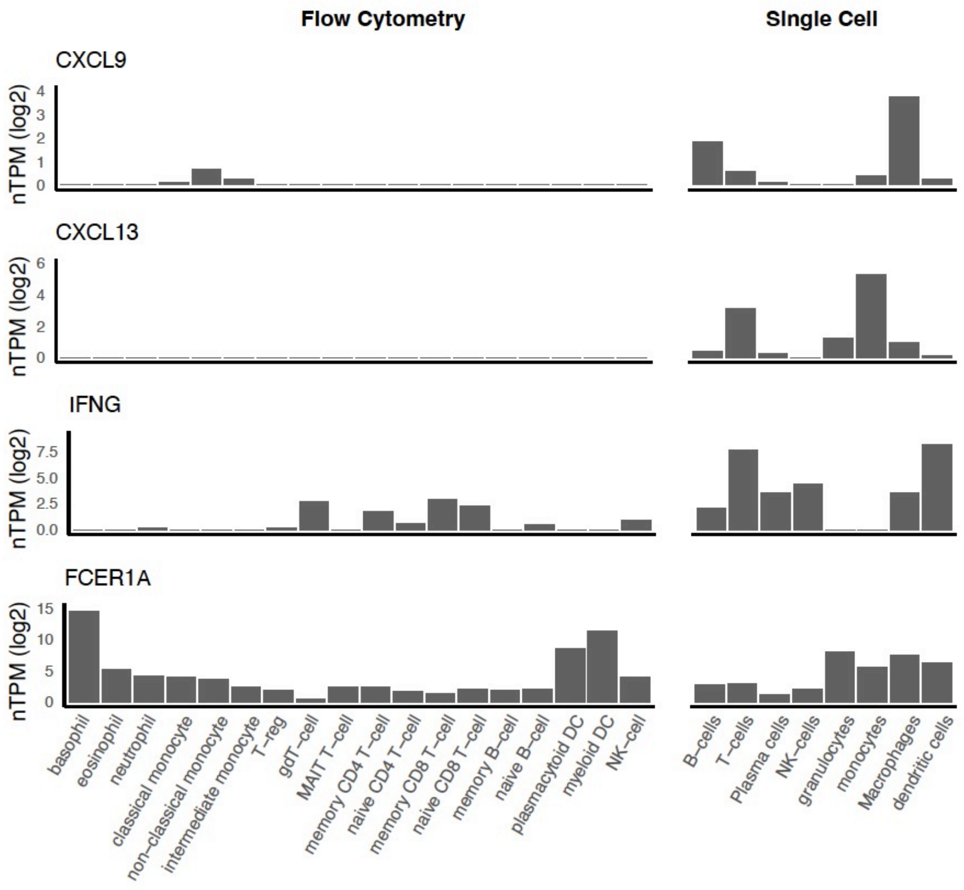

Supplementary Figure 5. **Expression of top differentially expressed genes in immune cell subtypes.** Normalized expression of 4 representative stromal biomarkers in different immune cell types. Immune cell expression data is derived from single-cell and flow cytometry sorted RNA-seq experiments from the Human Protein Atlas.
