# Supplementary material for: Transcriptome Deconvolution Reveals Absence of Cancer Cell Expression Signature in Immune Checkpoint Blockade Response
Source: Cancer Res Commun. 2024 Jun 26;4(6):1581–96. doi: 10.1158/2767-9764.CRC-23-0442 (PMC11203396; doi:10.1158/2767-9764.CRC-23-0442)
Supplement: Supplementary Figure 6 — Differentially enriched immune cell types between responders and non-responders. [file crc-23-0442-s06.pdf]

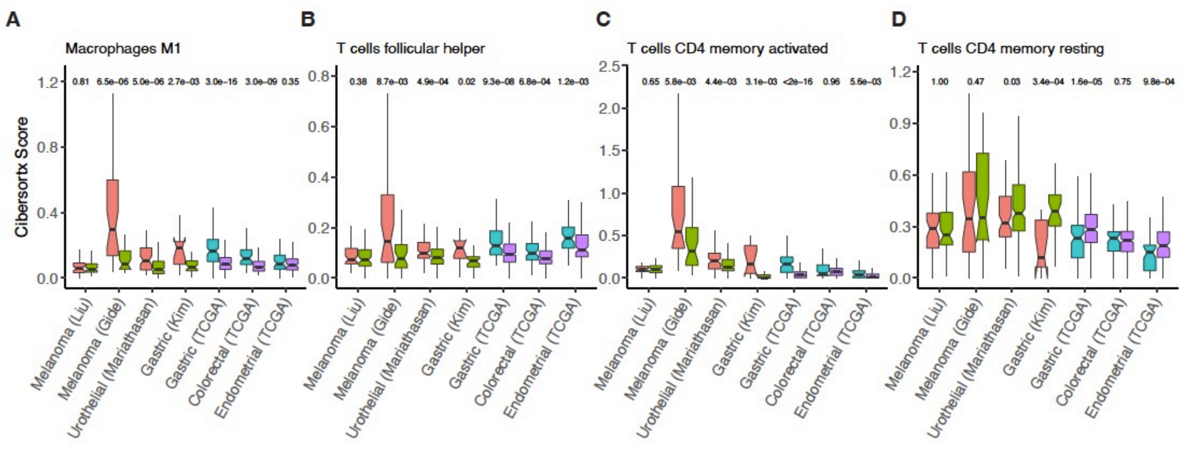

Supplementary Figure 6. **Differentially enriched immune cell types between responders and non-responders.** Boxplots showing the CIBERSORTx scores of 4 cell types that are significantly different between responders (MSI) and non-responders (MSS).
