# Supplementary material for: Transcriptome Deconvolution Reveals Absence of Cancer Cell Expression Signature in Immune Checkpoint Blockade Response
Source: Cancer Res Commun. 2024 Jun 26;4(6):1581–96. doi: 10.1158/2767-9764.CRC-23-0442 (PMC11203396; doi:10.1158/2767-9764.CRC-23-0442)
Supplement: Supplementary Figure 7 — Correlations between the abundance of immune cell subtypes with the gene expression of top stromal biomarkers in individual cohorts. [file crc-23-0442-s07.pdf]

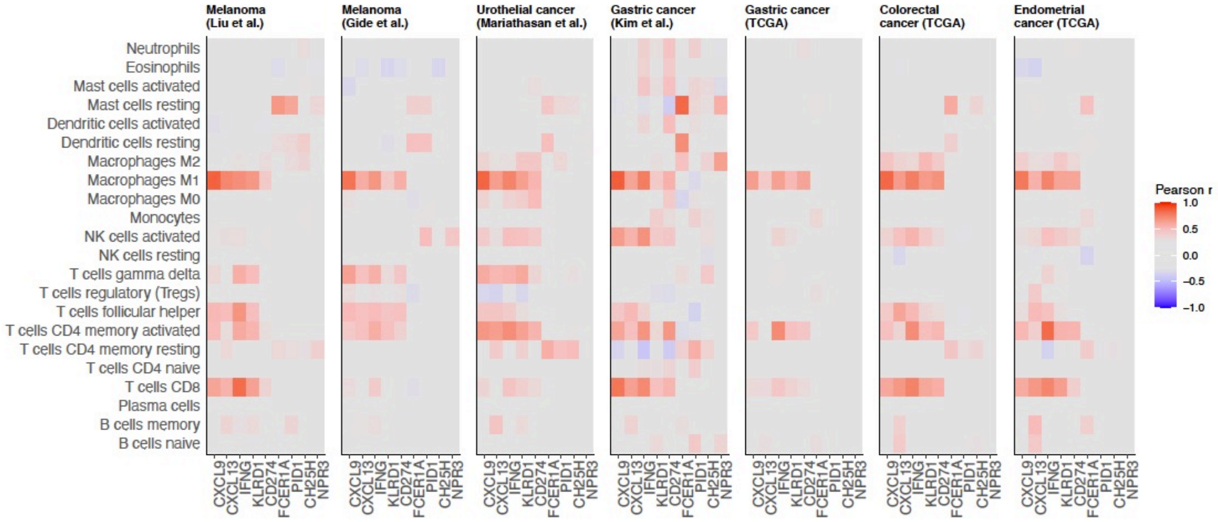

Supplementary Figure 7. Correlations between the abundance of immune cell subtypes with the gene expression of top stromal biomarkers in individual cohorts.
