# Supplementary material for: Transcriptome Deconvolution Reveals Absence of Cancer Cell Expression Signature in Immune Checkpoint Blockade Response
Source: Cancer Res Commun. 2024 Jun 26;4(6):1581–96. doi: 10.1158/2767-9764.CRC-23-0442 (PMC11203396; doi:10.1158/2767-9764.CRC-23-0442)
Supplement: Supplementary Figure 8 — Area under the receiver operating curve (AUC) of the cancer-DEG model on 6 test datasets. [file crc-23-0442-s08.pdf]

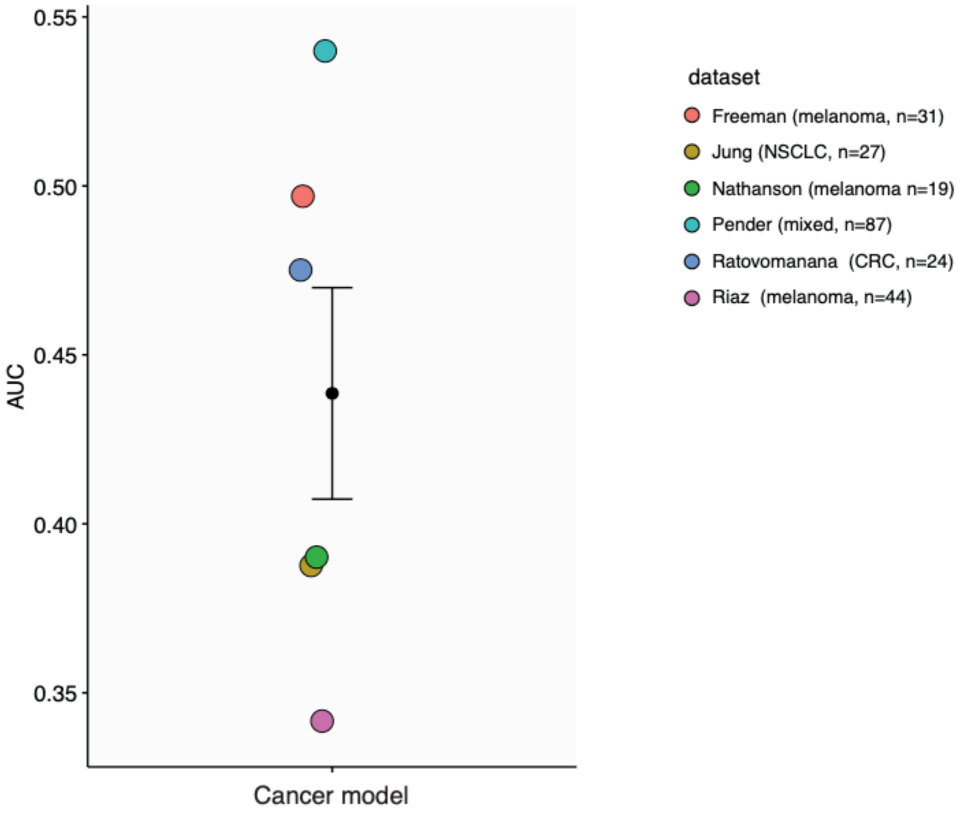

Supplementary Figure 8. Area under the receiver operating curve (AUC) of the cancer-DEG model on 6 test datasets. Black dot shows the mean AUC, and the error bars represent the standard error. Using one-sample t-test, we found that the predictive power of the model is not significantly different from the random expectation of AUC=0.5 (mean AUC= 0.44, p-value=0.11).
