# Supplementary material for: Transcriptome Deconvolution Reveals Absence of Cancer Cell Expression Signature in Immune Checkpoint Blockade Response
Source: Cancer Res Commun. 2024 Jun 26;4(6):1581–96. doi: 10.1158/2767-9764.CRC-23-0442 (PMC11203396; doi:10.1158/2767-9764.CRC-23-0442)
Supplement: Supplementary Figure 9 [file crc-23-0442-s09.pdf]

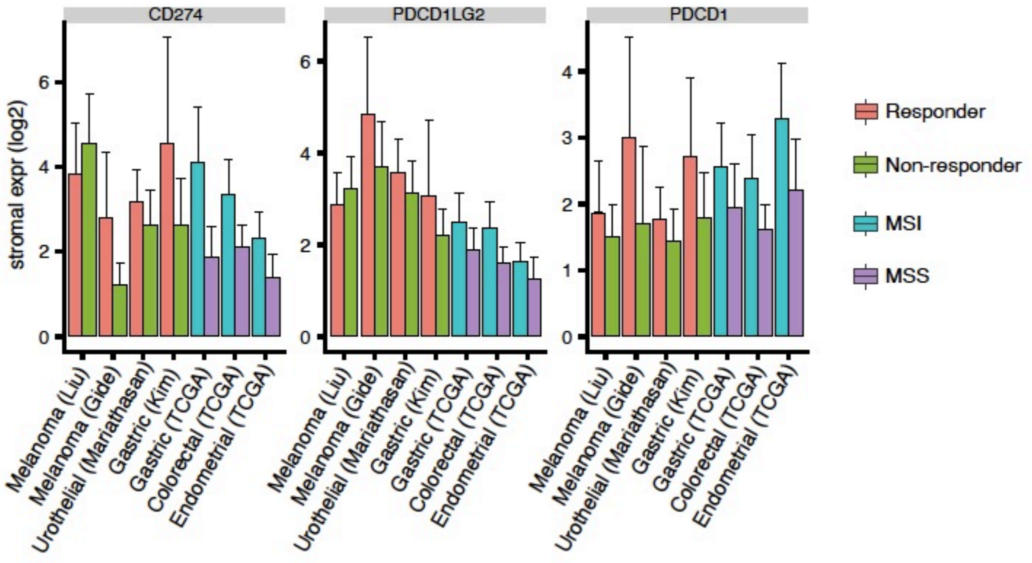

Supplementary Figure 9. **Differential expression of the PD1 receptor and its ligands PD-L1 and PD-L2 in the stroma.** Bar plots of deconvoluted stromal expression of the PD1 (*PDCD1*) receptor and its ligands PD-L1 (*CD274*) and PD-L2 (*PDCD1LG2*). Error bars represent estimated standard error.
