# Supplementary material for: Transcriptome Deconvolution Reveals Absence of Cancer Cell Expression Signature in Immune Checkpoint Blockade Response
Source: Cancer Res Commun. 2024 Jun 26;4(6):1581–96. doi: 10.1158/2767-9764.CRC-23-0442 (PMC11203396; doi:10.1158/2767-9764.CRC-23-0442)
Supplement: Supplementary Figure 10 — Association between model prediction and overall survival. [file crc-23-0442-s10.pdf]

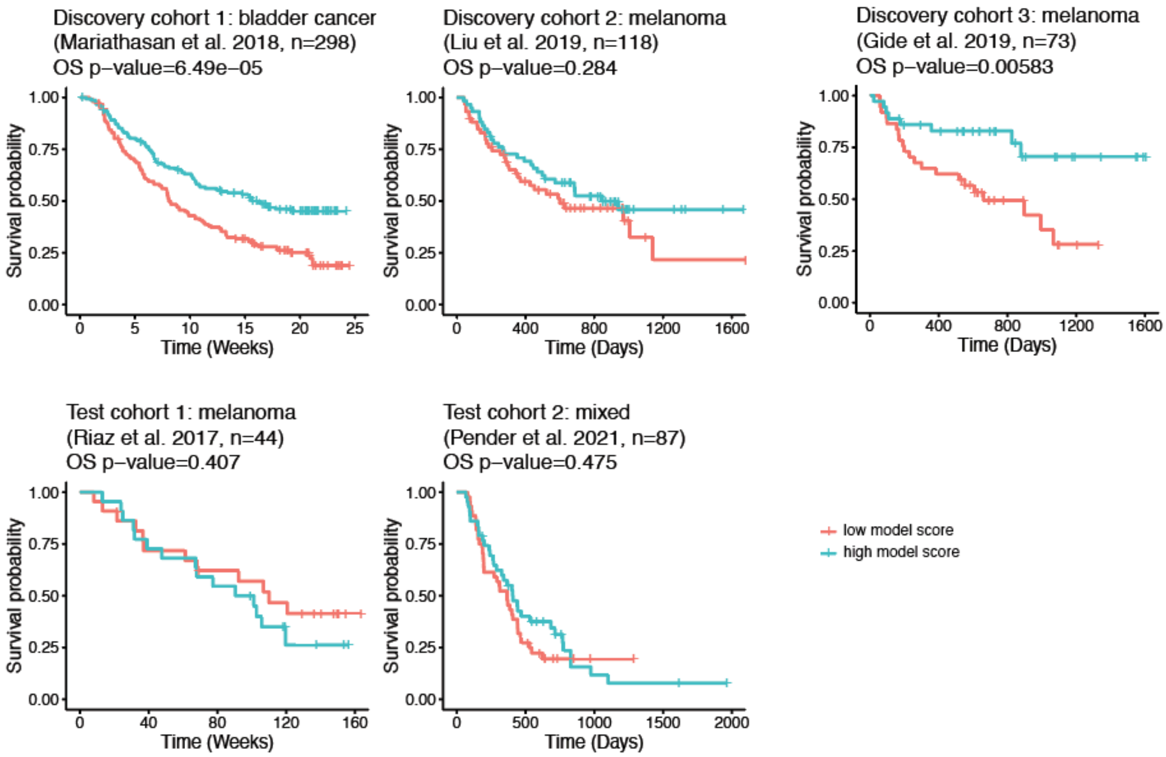

Supplementary Figure 10. **Association between model prediction and overall survival.** Kaplan-Meier curves of overall free survival for patients with high vs low predicted scores (greater than or less than median); p-values from log-rank test shown.
