# Supplementary material for: Transcriptome Deconvolution Reveals Absence of Cancer Cell Expression Signature in Immune Checkpoint Blockade Response
Source: Cancer Res Commun. 2024 Jun 26;4(6):1581–96. doi: 10.1158/2767-9764.CRC-23-0442 (PMC11203396; doi:10.1158/2767-9764.CRC-23-0442)
Supplement: Supplementary Table 1 — Clinical characteristics of the study cohorts. [file crc-23-0442-s11.pdf]

						Response		
**cohort**	**type**	**PMID**	**cancer type**	**ICB agent**	**Type of ICB**	**R**	**NR**	**total**
Mariathasan	discovery	29443960	bladder	atezolizumab	anti-PD-L1	68	230	298
Kim	discovery	30013197	gastric	pembrolizumab	anti-PD-1	12	33	45
Liu	discovery	31792460	melanoma	nivolumab or pembrolizumab	anti-PD-1	47	71	118
Gide	discovery	30753825	melanoma	pembrolizumab or nivolumab alone, or with ipilimumab	anti-PD-1 alone or with anti-CTLA-4	40	33	73
Riaz	validation	29033130	melanoma	nivolumab	anti-PD-1	10	39	49
Pender	validation	33020056	lung (20), melanoma (18), breast (13), others (36)	not specified	PD-1/PD-L1 (57), CTLA-4 (3), combo (26), others (1)	15	72	87
Freeman	validation	35243413	melanoma	not specified	PD-1/PD-L1 (13), CTLA-4 (15), combo (3)	10	21	31
Jung	validation	31537801	lung	not specified	anti-PD-1 or PD-L1	19	8	27
Nathanson	validation	27956380	melanoma	ipilimumab	anti-CTLA-4	12	7	19
Ratovomanana	validation	37269904	colorectal	nivolumab alone or with ipilimumab	anti-PD-1 alone or with anti-CTLA-4	13	11	24

Supplementary Table 1. **Clinical characteristics of the study cohorts**
