# Supplementary material for: Transcriptome Deconvolution Reveals Absence of Cancer Cell Expression Signature in Immune Checkpoint Blockade Response
Source: Cancer Res Commun. 2024 Jun 26;4(6):1581–96. doi: 10.1158/2767-9764.CRC-23-0442 (PMC11203396; doi:10.1158/2767-9764.CRC-23-0442)
Supplement: Supplementary Table 3 — Data source of the ICB cohort. [file crc-23-0442-s13.pdf]

cohort	Data source	PMID
Mariathasan	Supplementary website http://research-pub.gene.com/IMvigor210CoreBiologies/	29443960
Kim	European Nucleotide Archive under accession PRJEB25780	30013197
Liu	Supplementary Table 2	31792460
Gide	European Nucleotide Archive under accession PRJEB23709	30753825
Riaz	Gene Expression Omnibus under accession GSE91061	29033130
Pender	Supplementary website https://www.bcgsc.ca/downloads/immunoPOG/	33020056
Freeman	Supplementary Table 4	35243413
Jung	Gene Expression Omnibus under accession GSE135222	31537801
Nathanson	Supplementary Data	27956380
Ratovomanana	Supplementary website https://github.com/CRSA-MSI/R-ICI_MNB_Survival	37269904

Supplementary Table 3. Data source of the ICB cohorts
